# Genome-Wide Association Mapping Reveals Multiple QTLs Governing Tolerance Response for Seedling Stage Chilling Stress in *Indica* Rice

**DOI:** 10.3389/fpls.2017.00552

**Published:** 2017-04-25

**Authors:** Elssa Pandit, Swaleha Tasleem, Saumya R. Barik, Durga P. Mohanty, Deepak K. Nayak, Shakti P. Mohanty, Sujata Das, Sharat K. Pradhan

**Affiliations:** Crop Improvement Division, Central Rice Research Institute (ICAR)Cuttack, India

**Keywords:** seedling stage chilling tolerance, association mapping, genetic diversity, population structure, linkage disequilibrium

## Abstract

Rice crop is sensitive to cold stress at seedling stage. A panel of population representing 304 shortlisted germplasm lines was studied for seedling stage chilling tolerance in *indica* rice. Six phenotypic classes were exposed to six low temperature stress regimes under control phenotyping facility to investigate response pattern. A panel of 66 genotypes representing all phenotypic classes was used for ensuring genetic diversity, population structure and association mapping for the trait using 58 simple sequence repeat (SSR) and 2 direct trait linked markers. A moderate level of genetic diversity was detected in the panel population for the trait. Deviation of Hardy-Weinberg's expectation was detected in the studied population using Wright's F statistic. The panel showed 30% variation among population and 70% among individuals. The entire population was categorized into three sub-populations through STRUCTURE analysis. This revealed tolerance for the trait had a common primary ancestor for each sub-population with few admix individuals. The panel population showed the presence of many QTLs for cold stress tolerance in the individuals representing like genome-wide expression of the trait. Nineteen SSR markers were significantly associated at chilling stress of 8°C to 4°C for 7–21 days duration. Thus, the primers linked to the seedling stage cold tolerance QTLs namely qCTS9, qCTS-2, qCTS6.1, qSCT2, qSCT11, qSCT1a, qCTS-3.1, qCTS11.1, qCTS12.1, qCTS-1b, and CTB2 need to be pyramided for development of strongly chilling tolerant variety.

## Introduction

Rice is life for millions of people in the globe. Globally, rice the most important food crop for 3.5 billion populations with a total production of 715 million tons (FAO, 2013). Rice cultivation covers around 160 million hectares on the earth. In India, around one-fourth (43 million hectares) of the world's rice area is cultivated for rice with a total production of 104.8 million tons (DAC, 2015). Globally, around 15 million hectares of rice fields are affected by cold weather spreading in 24 different countries. Currently, there is a ceiling of rice yield potential which is not sufficient to feed the ever growing population. Therefore, it is essential to increase the rice production even from abiotic stressful rice-growing ecosystems to enable the increasing food demand to commensurate with increasing population. Around 4 million hectares of rice areas comprising boro and part of dry season rice of India are highly affected by seedling-stage cold causing delay in growth of the plant which subsequently further aggravated as it coincides with high temperature during flowering stage (Pradhan et al., 2016). The ideal temperature for rice germination and seedling growth is 25–35°C and the temperature below 15°C normally affects various processes (Nakagahra et al., 1997). Rice plants at early vegetative stage are sensitive to cold stress resulting in poor germination, slow early growth, yellowing and withering of plant, reduced tillering, and stunted growth which finally decrease in overall yield (Zhang et al., 2005; Andaya and Tai, 2006; Lou et al., 2007; Suh et al., 2010; Pradhan et al., 2015). The extent of cold and high temperature stress is increasing due to global climate change. For instance, in Asia sudden low temperature and high temperature stress covering areas have been widened (Pradhan et al., 2015, 2016). Therefore, stress tolerance QTLs for low and high temperature stress need to be stacked in the high yielding varieties for cultivation in the target environments.

The inheritance of cold tolerance in rice is complex in nature and mechanism of the tolerance is difficult to explain using single or few genes. Gene mapping studies using various populations have detected role of many single genes and QTLs controlling cold stress tolerance during seedling stage of rice. More than 30 QTLs are reported to be involved in tolerance response for the trait in rice and they are present on different chromosomal locations (Kwak et al., 1984; Nagamine, 1991; Kim et al., 2000, 2014; Misawa et al., 2000; Qian et al., 2000; Andaya and Mackill, 2003; Qu et al., 2003; Fujino et al., 2004; Zhan et al., 2005; Zhang et al., 2005; Andaya and Tai, 2006; Jiang et al., 2006, 2008; Han et al., 2007; Lou et al., 2007; Koseki et al., 2010; Wang et al., 2011; Suh et al., 2012). Pyramiding of these QTLs for cold tolerance breeding program is difficult due to more in numbers governing the tolerance response, lack of information on robust molecular markers and highly tolerant donors containing many QTLs for marker-assisted breeding. Screening and identification of highly tolerant donors for seedling stage cold tolerance from the germplasm pool is an important step for selection of donor parents in cold tolerance breeding program. Molecular markers, usually SSR or Simple Sequence Length Polymorphism (SSLP) are commonly being used for assessing rice genetic diversity (Panaud et al., 1996; Wu and Tanksley, 1996; Xiao et al., 1996; Olufowote et al., 1997; Thanh et al., 1999; Herrera et al., 2008; Pervaiz et al., 2010; Das et al., 2013; Babu et al., 2014; Anandan et al., 2016; Pradhan et al., 2016). Hence, genetic diversity available in parental lines should be assessed by using the trait linked SSRs and gene specific markers for seedling stage chilling stress tolerance before selection of parental lines. The genetic diversity and structure of the population for the trait of concern need to be studied for association mapping which could be useful in molecular breeding programs. For detecting a perfect marker-phenotype association, the population should not show spurious association or unequal relatedness within the population (Jagadish et al., 2007). Therefore, population structure (*Q*) with relative kinship (*K*) matrics analyses is used to check and correct the panel population composition for linkage disequilibrium (LD) mapping analyses (Yu et al., 2006). Thus, marker-based mixed linear model for better kinship estimate is considered appropriate for association mapping approaches that have shown to perform better than other model analysis.

Most of previous papers reported QTLs for the trait were derived through bi-parental mapping populations and mainly in *japonica* rice background. Bi-parental mapping is much resource consuming and very less number of alleles can be identified taking long time period with less resolution (Cardon and Bell, 2001; Flint-Garcia et al., 2003; Stich et al., 2005; Roy et al., 2006; Pradhan et al., 2016). These problems are eliminated or reduced by association mapping or linkage disequilibrium (LD) mapping approach. The purpose of LD mapping is to estimate the correlations between genotypes and desired phenotypic trait in a panel population containing genotypes from all the phenotypic classes. Association mapping, also known as linkage disequilibrium mapping, utilizes allelic variation in natural populations, and is capable of identifying many loci simultaneously for multiple traits (Flint-Garcia et al., 2005). The association mapping in rice has been reported for various traits like grain yield components (Agrama et al., 2007), agronomic traits (Huang et al., 2010), agro-morphologic and yield traits (Zhao et al., 2011), tillering stage cold tolerance (Zhang et al., 2012), flowering and grain yield traits (Huang et al., 2012), grain quality traits (Zhao et al., 2013), drought tolerance traits (Muthukumar et al., 2015), salinity tolerance (Kumar et al., 2015), cold tolerance at germination and booting stages (Pan et al., 2015) mineral element in grain (Huang et al., 2015), early seedling vigor (Anandan et al., 2016), and high temperature stress tolerance (Pradhan et al., 2016). However, there is no such report till now for QTLs or genes for seedling stage cold tolerance through association analysis in *indica* rice. Therefore, in this study, a set of 304 shortlisted genotypes comprising mainly rice cultivars of high-elevation regions of India along with tolerant genotypes of other places of the country were analyzed for genetic diversity, population structure and association mapping using 60 linked markers for the trait. The robust markers showing strong effects and the identified highly tolerant donors could be the potential donor parents and markers in developing cultivars with seedling stage cold tolerance by molecular marker-assisted selection.

## Materials and methods

### Plant materials and field screening

The shorted materials comprising of 304 germplasm lines were pooled for the study from ICAR-National Rice Research Institute, Cuttack (NRRI) gene bank. Majority of the collection were from cold and higher elevation regions of the country including cold tolerant lines of NRRI (Table S1). The accessions were sown during December, 2014 in plots arranged in an augmented block design placing four check varieties (KalingaIII, Sahabhagidhan, Annada, and Vandana). A population size of three rows per germplasm line with a row length of 4 m and spacing of 20 × 15 cm were followed during sowing of seeds. Daily average minimum temperature was recorded during seedling stage of the crop. Scoring of germplasm lines were done during the very low temperature period and recorded the scores for the change in leaf color, leaf rolling and plant growth. Screening of the germplasms for cold tolerance was done following IRRI-SES score (IRRI, 2014).

### Screening under control condition

A panel of 66 genotypes representing all phenotypic classes were direct seeded in partially soil filled pots and uniform growth was maintained under green house chamber of RGA-cum-Phytotron of the Institute for phenotyping chilling stress tolerance under controlled condition during June-July, 2015 (Table 1). Genotypes were screened following the published protocol for chilling stress tolerance (Pradhan et al., 2015). As per the protocol, seedlings were grown in RGA-cum-Phytotron till three-leaf stage set at 25°C temperature and photoperiod of 12 h. At this stage, the weak seedlings were removed and healthy seedlings were exposed to low temperature regime (LTR) treatments in the growth chamber with a setting of 75–85% relative humidity and 800 μ moles s^−1^ m^−2^ light intensity. The low temperature regime1 (LTR1) was by exposing the seedling to a temperature of 25°C for 3 days; low temperature regime 2 (LTR2) with a gradual decrease in temperature to 15°C from 25°C and maintained at this temperature for 7 days; low temperature regime 3 (LTR3) with a gradual decrease in temperature to 8°C from 15°C and maintained at this temperature for 7 days; low temperature regime 4 (LTR4) with a gradual decrease in temperature to 4°C from 8°C and maintained at this temperature for 7 days; low temperature regime 5 (LTR5) with an exposure temperature of 4°C for 14 days and low temperature regime 6 (LTR6) with an exposure temperature of 4°C for 21 days. Complete randomized designs with two replications were followed for the experiment. The genotypes were evaluated using a modified IRRI-SES score under control facility. The modified score was given for field screening with miner modification as follows. 1 = Seedlings dark green; 3 = Seedling light green; 5 = Seedling with rolled leaves and light green to brownish yellow in color; 7 = Seedling with rolled leaves and brownish green in color; 9 = Seedling with rolled leaves with brown in color.

**Table 1:** **Average score for seedling stage cold tolerance of 66 rice genotypes under cold growth chamber at different temperature regimes**.

**Sl. no**.	**Genotype name**	**Mean SES score of genotype after exposure (days) to various low temperature regimes**						**Response to chilling stress tolerance**
		**3 days at 25°C**	**7 days at 15° C**	**7 days at 8°C**	**7 days at 4°C**	**14 days at 4°C**	**21 days at 4°C**	
		**SES score**	**SES score**	**SES score**	**SES score**	**SES score**	**SES score**	
1	KalingaIII	1 (100)	1 (100)	1 (97.5)	1 (95)	1 (92.5)	7 (85)	HT
2	Geetanjali	1 (100)	1 (100)	1 (100)	1 (100)	1 (95)	3 (87.5)	VHT
3	Sahabhagi dhan	1 (97.5)	7 (60)	7 (97.5)	9 (100)	9 (100)	9 (100)	HS
4	Govind	1 (100)	1 (92.5)	1 (82.5)	3 (85)	5 (67.5)	9 (97.5)	HT
5	Ajaya	1 (100)	1 (92.5)	1 (85)	3 (77.5)	3 (77.5)	9 (97.5)	HT
6	Satabdi	1 (100)	1 (90)	5 (77.5)	7 (75)	9 (95)	9 (100)	MT
7	Krishnahansa	1 ()	1 (92.5)	1 (85)	3 (70)	7 (65)	9 (100)	T
8	Kamesh	1 (100)	1 (90)	1 (90)	1 (85)	3 (77.5)	9 (100)	HT
9	Vandana	1 (100)	1 (90)	5 (77.5)	9 (95)	9 (100)	9 (100)	MT
10	Paun	1 (100)	1 (90)	1 (85)	1 (87.5)	3 (77.5)	9 (97.5)	HT
11	Radang	1 (97.5)	1 (95)	1 (90)	3 (80)	5 (72.5)	9 (97.5)	HT
12	Manipuri dhan	1 (95)	1 (90)	1 (87.5)	1 (87.5)	3 (80)	9 (100)	HT
13	Rungpchi	1 (95)	1 (95)	1 (87.5)	1 (87.5)	1 (82.5)	7 (95)	HT
14	MR37 (Farmer selection)	1 (100)	1 (95)	1 (90)	3 (80)	5 (75)	9 (92.5)	HT
15	Umleng-2	1 (100)	1 (90)	1 (92.5)	1 (85)	3 (80)	9 (95)	HT
16	Langma	1 (100)	1 (97.5)	1 (95)	1 (92.5)	1 (90)	5 (87.5)	VHT
17	Charmui	1 (97.5)	1 (95)	1 (90)	3 (80)	5 (77.5)	9 (95)	HT
18	Jamak	1 (97.5)	1 (92.5)	1 (90)	1 (85)	3 (85)	9 (100)	HT
19	Umleng-1	1 (100)	1 (95)	1 (87.5)	1 (90)	1 (92.5)	5 (92.5)	VHT
20	Langme-1	1 (100)	1 (95)	1 (90)	3 (80)	5 (65)	9 (95)	HT
21	Mopu	1 (95)	1 (92.5)	1 (92.5)	1 (90)	3 (85)	9 (97.5)	HT
22	Tazek	1 (97.5)	1 (95)	1 (90)	1 (92.5)	1 (87.5)	7 (90)	HT
23	Umbo	1 (97.5)	1 (95)	1 (92.5)	1 (90)	3 (85)	9 (97.5)	HT
24	Serum	1 (95)	1 (90)	1 (90)	1 (87.5)	3 (80)	9 (97.5)	HT
25	Bamak	1 (92.5)	1 (95)	1 (87.5)	3 (85)	5 (70)	9 (97.5)	HT
26	Lagmin	1 (97.5)	1 (95)	1 (92.5)	1 (90)	3 (87.5)	9 (95)	HT
27	Tabadugu	1 (100)	1 (92.5)	1 (90)	5 (82.5)	7 (90)	9 (92.5)	T
28	Langme-2	1 (100)	1 (95)	1 (87.5)	3 (80)	5 (70)	9 (97.5)	HT
29	Itanagar dhan	1 (97.5)	1 (95)	1 (90)	1 (82.5)	3 (80)	9 (97.5)	HT
30	Phouurel	1 (92.5)	3 (65)	7 (87.5)	9 (97.5)	9 (100)	9 (100)	MS
31	Chakhaopspoireitol	1 (92.5)	1 (85)	5 (75)	7 (82.5)	9 (100)	9 (100)	MT
32	Phourel Angoubi	1 (95)	1 (85)	1 (65)	7 (85)	9 (97.5)	9 (100)	MT
33	Phourelamubi	1 (92.5)	1 (85)	1 (67.5)	7 (77.5)	9 (95)	9 (100)	MT
34	Phougang	1 (92.5)	1 (90)	3 (70)	7 (80)	9 (97.5)	9 (100)	MT
35	Phoungang	1 (95)	1 (92.5)	3 (82.5)	7 (85)	9 (95)	9 (100)	MT
36	Langphou phougang	1 (95)	1 (77.5)	1 (72.5)	7 (80)	9 (95)	9 (100)	MT
37	Changphoi	1 (97.5)	1 (92.5)	3 (77.5)	7 (85)	7 (97.5)	9 (100)	MT
38	Uteibi	1 (95)	1 (90)	3 (75)	7 (85)	9 (97.5)	9 (100)	MT
39	Khangkuailang	1 (92.5)	1 (82.5)	1 (70)	7 (77.5)	9 (95)	9 (100)	MT
40	Thingjangra	1 (92.5)	1 (80)	3 (92.5)	7 (75)	9 (97.5)	9 (100)	MT
41	Photem	1 (90)	1 (82.5)	7 (90)	9 (77.5)	9 (97.5)	9 (100)	MT
42	9426	1 (87.5)	1 (87.5)	7 (85)	9 (95)	9 (97.5)	9 (100)	MS
43	9428	1 (95)	3 (87.5)	7 (87.5)	9 (97.5)	9 (100)	9 (100)	MS
44	Langmenti	1 (97.5)	1 (75)	3 (80)	7 (85)	9 (100)	9 (100)	MT
45	Aujari	1 (95)	1 (80)	3 (70)	7 (80)	9 (97.5)	9 (100)	MT
46	Japan phou	1 (92.5)	1 (77.5)	1 (72.5)	7 (85)	9 (95)	9 (100)	MT
47	Mayang khang	1 (95)	1 (75)	3 (90)	9 (85)	9 (98)	9 (100)	MT
48	Napdai	1 (97.5)	1 (75)	7 (85)	9 (95)	9 (100)	9 (100)	MS
49	Changlei-1	1 (95)	3 (85)	9 (95)	9 (92.5)	9 (100)	9 (100)	MS
50	Changlei-2	1 (100)	1 (90)	5 (75)	7 (70)	7 (97.5)	9 (100)	MT
51	Sangsangba-1	1 (95)	1 (92.5)	5 (80)	7 (75)	7 (97.5)	9 (100)	MT
52	Phourel angab	1 (97.5)	1 (90)	5 (82.5)	7 (72.5)	7 (97.5)	9 (100)	MT
53	Sangsangba-2	1 (97.5)	1 (90)	5 (90)	7 (77.5)	7 (95)	9 (100)	MT
54	CR 143-2-2	1 (100)	1 (82.5)	5 (85)	7 (90)	9 (97.5)	9 (100)	MT
55	Sadabahar	1 (97.5)	3 (82.5)	7 (90)	9 (87.5)	9 (97.5)	9 (100)	MS
56	Tejaswini	1 (97.5)	5 (90)	7 (77.5)	9 (85)	9 (100)	9 (100)	MS
57	Vanaprava	1 (100)	5 (72.5)	7 (82.5)	9 (85)	9 (100)	9 (100)	MS
58	Swarnaprava	1 (100)	5 (75)	7 (80)	9 (97.5)	9 (100)	9 (100)	MS
59	Virendra	1 (95)	3 (85)	5 (67.5)	9 (92.5)	9 (97.5)	9 (100)	MT
60	Swarna	1 (100)	5 (82.5)	7 (77.5)	9 (100)	9 (100)	9 (100)	MS
61	Tapaswini	1 (92.5)	5 (85)	7 (90)	9 (100)	9 (100)	9 (100)	MS
62	Gayatri	1 (95)	5 (80)	7 (87.5)	9 (100)	9 (100)	9 (100)	MS
63	Naveen	1 (92.5)	5 (75)	7 (85)	9 (100)	9 (100)	9 (100)	MS
64	IR-64	1 (95)	1 (95)	3 (77.5)	7 (80)	9 (95)	9 (100)	MT
65	Pratikshya	1 (97.5)	5 (77.5)	7 (90)	7 (100)	9 (100)	9 (100)	MS
66	Ranidhan	1 (95)	5 (80)	7 (87.5)	9 (100)	9 (100)	9 (100)	MS
CV%		4.21	9.72	10.64	11.73	11.21	5.7	

*Parentheses contains % of plants of a genotype affected due to seedling stage cold stress. SES, Standard Evaluation system; HS, Highly susceptible; MS, Moderately susceptible; MT, Moderately tolerant; T, Tolerant and HT, Highly Tolerant; VHT, Very highly tolerant*.

### DNA isolation and selection of SSR markers

Leaf samples were collected from 2 week old seedlings of 66 genotypes present in the panel population. Total genomic DNA of all the studied genotypes were isolated using liquid nitrogen for grinding using CTAB extraction buffer (100 mM Tris-HCl pH 8, 20 mM EDTA pH 8, 1.3M NaCl, 2% CTAB) and chloroform-Isoamyl alcohol extraction followed by RNAase treatment and ethanol precipitation (Murray and Thompson, 1980). DNA concentration was estimated by using agarose gel electrophoresis. The sample DNA was diluted to ~30 ng/μL. Earlier published reports on bi-parental mappings were used to select the seedling stage cold tolerance markers (58 linked SSR and 2 direct) and the distribution of markers covered all the chromosomes to illustrate the diversity.

### PCR amplification and visualization of markers linked to seedling stage chilling stress

Polymerase chain reaction was performed by taking 20 μl aliquot using 1.5 mM Tris HCL (pH 8.75), 50 mM KCL, 2 mM MgCl_2_, 0.1% TrotonX-100, 200 μM each of dATP, dCTP, dTTP, dGTP, 4 pmole of each forward and reverse primers (Table 2), 1 unit of Taq polymerase and 30 ng of genomic DNA. A Programmable Thermal Cycler was used for amplification of genomic DNA samples (Veriti, Applied BioSciences). First, the reaction mixture was denatured for 4 min at 94°C and then continued to 35 cycles of 1 min denaturation at 94°C, 1 min annealing at 55°C, 1 min extension at 72°C, and then a final extension for 10 min at 72°C. Agarose gel of 2.5% containing 0.8 μg/ml Ethidium Bromide was used for electrophoresis. Aliquots of 10 μl of the products from PCR amplification were loaded in the agarose gel. Size of amplicons was determined by using 50 bp DNA ladder. The gel was run at 60 volts (2.5 V/cm) in 1X TBE (pH 8.0) for 4 h and photographed using a Gel-Doc System (SynGene).

**Table 2:** **Information on the selected 60 linked molecular markers used for seedling stage chilling stress tolerance in *indica* rice**.

**Sl. no**.	**Marker name**	**QTLs**	**Chr. no**.	**Distance**	**Phenotypic variance %**	**Expected band size (bp)**	**Repeat motif**	**References**
1	RM 1347	qSCT10	10	17 cM	6.1	119	(AG)23	Kim et al., 2014
2	RM 328	qCTS9	9	82.4 cM		172	(CAT)5	Long-zhi et al., 2004
3	RM 152	qCTS-8	8	9.4 cM	7.4	151	(GGC)10	Lou et al., 2007
4	RM 341	qCTS-2	2	82.7 cM	27.42	172	(CTT)20	Lou et al., 2007
5	RM 50	qCTS6-1	6	3.8 Mb	15.3	201	(CTAT)4(CT)15	Andaya and Mackill, 2003
6	RM2634	qSCT2	2	15.6 cM	6.5	154	(AT)31	Kim et al., 2014
7	RM4112	qSCT11	11	23.1 cM	16.5	153	(TA)14	Kim et al., 2014
8	RM5310	qSCT1a	1	11.3 cM	10.6	154	(TC)12	Kim et al., 2014
9	RM7179	qCTS-3.1	3	35.3 Mb	15.7	165	(ATAG)6	Wang et al., 2011
10	RM3701	qCTS11.1	11	63 Mb	9	174	(GA)15	Wang et al., 2011
11	RM104	qCTS12.1	1	186.6 cM	–	222	(GA)9	Long-zhi et al., 2004
12	RM9	qCTS-1b	1	92.4 cM	11.31	136	(GA)15GT(GA)3	Lou et al., 2007
13	RM1211	qSCT2	2	15.6 cM	6.5	213	(AG)14	Kim et al., 2014
14	RM 3375A	qCTS-4a	4	5.9 Mb	8.3	186	(CT)16	Suh et al., 2012
15	RM 5746	qCSH10	12	–	–	176	(ACG)8	Pradhan et al., 2015
16	RM 286	qCSH10	11	0–0 cM	–	110	(GA)16	Pradhan et al., 2015
17	RM 84	qCTS-1a	1	26.2 cM	6.46	113	(TCT)10	Lou et al., 2007
18	RM 561	qCTS-2	2	74.1 cM	27.42	190	(GA)11	Lou et al., 2007
19	RM 253	qCTS6-1	6	3.8 Mb	15.3	141	(GA)25	Andaya and Mackill, 2003
20	RM284	qCSH10	10	2.3 Mb	10.4	141	(GA)8	Andaya and Mackill, 2003
21	RM 239	qCSH10	10	2.3 Mb	10.4	144	(AG)5TG(AG)2	Andaya and Mackill, 2003
22	RM256	qCTS12.1	1	101.5 cM	–	127	(CT)21	Long-zhi et al., 2004
23	RM1812	qCSH10	11	–	–	136	(AT)16	Pradhan et al., 2015
24	RM558	qCTS-4a	4	5.9 Mb	8.3	246	(ATTG)5	Suh et al., 2012
25	RM173	qCTS5	5	99.8cM	–	186	(GA)9	Long-zhi et al., 2004
26	RM 14978	qCTB2	4	24.57 cM	24.57	225	(TC)10	Bonnecarrere et al., 2015
27	RM14960	qCTB2	4	24.09 cM	24.09	192	(CGA)7	Bonnecarrere et al., 2015
28	RM6651	qCTB2	4	11.81 cM	11.81	134	(GTG)8	Bonnecarrere et al., 2015
29	RM22491	qCTS8.1	11	23.6 Mb	22.4	147	(CTC)7	Wang et al., 2011
30	RM590	qSCT10	10	17 cM	6.1	137	(TCT)10	Kim et al., 2014
31	In11-d1	qSCT11	11	Direct	–	158		Kim et al., 2014
32	RM 245	qCTS9	9	112.3cM	–	150	(CT)14	Long-zhi et al., 2004
33	RM 3602	qSCT1a	1	11.3 cM	10.6	120	(GA)13	Kim et al., 2014
34	RM 493	qCTS-1b	1	79.7 cM	11.31	211	(CTT)9	Lou et al., 2007
35	RM13335	qCTB2	4	24.52 cM	24.52	168	(AG)21	Bonnecarrere et al., 2015
36	RM282	qCTS-3.1	3	41.7 Mb	15.7	136	(GA)15	Wang et al., 2011
37	RM 5704	qCTS11.1	11	55.4 Mb	9	210	(AAT)20	Wang et al., 2011
38	In1-c3	qSCT1	1	Direct	–	241	–	Kim et al., 2014
39	RM1113	qSCT4	4	14.8 cM	12	150	(AG)12	Kim et al., 2014
40	RM 3648	qCTS-4b	4	8.8 Mb	7.8	186	(GA)14	Suh et al., 2012
41	RM 85	qCTS3	3	9 Mb	13.8	107	(TGG)5(TCT)12	Andaya and Mackill, 2003
42	RM 472	qCTS-1c	1	171.6 cM	8.81	296	(GA)21	Lou et al., 2007
43	RM 297	qCTS1	1	5.9 Mb	9.3	148	(GA)13	Andaya and Mackill, 2003
44	RM 1341	qSCT11	11	23.1cM	16.5	183	(AG)22	Kim et al., 2014
45	RM 506	qCTS-8	8	0-0 cM	7.4	123	(CT)13	Lou et al., 2007
46	RM 2799	qCTS-4b	4	8.8 Mb	7.8	156	(AT)35	Suh et al., 2012
47	RM 7003	qCSH10	12	–	–	101	(AAAC)6	Pradhan et al., 2015
48	RM305	qCTS5	5	86.9cM	–	203	(GT)4 + degener	Long-zhi et al., 2004
49	RM319	qCTS1	1	5.9 Mb	9.3	134	(GT)10	Andaya and Mackill, 2003
50	RM522	qCTS-1a	1	33.9 cM	6.46	143	(AAT)6	Lou et al., 2007
51	RM315	qCTS-1c	1	165.3cM	8.81	133	(AT)4(GT)10	Lou et al., 2007
52	RM200	qCTS3	3	9 Mb	13.8	122	(GA)16	Andaya and Mackill, 2003
53	RM6091	qCTS11.2	11	66.5 Mb	6.8	126	(CCT)11	Wang et al., 2011
54	RM6947	qCTS11.2	12	5.9 Mb	5.5	155	(TTC)8	Wang et al., 2011
55	RM6356	qCTS8.1	11	17.1 Mb	22.4	156	(GAA)10	Wang et al., 2011
56	RM26632	qCTS11.2	11	70.7 Mb	6.8	434	(TCTT)9	Wang et al., 2011
57	RM3739	qCTS12.1	12	5.9 Mb	5.5	110	(GA)16	Wang et al., 2011
58	RM6547	qCTB2	4	18.98 cM	18.98	165	(GCT)9	Bonnecarrere et al., 2015
59	RM11239	qCTB2	4	22.48 cM	22.48	440	(TG)15	Bonnecarrere et al., 2015
60	RM18776	qCTB2	4	22.31 cM	22.31	182	(GA)13	Bonnecarrere et al., 2015

### Genetic diversity, population structure, and linkage disequilibrium mapping

Data were scored on the basis of presence or absence of the alleles for each genotype-primer combination and arranged in a binary data matrix as discrete variables. PowerMarker Ver3.25 program was used for data analysis to generate number of alleles, allele frequency, gene diversity, heterozygosis, and polymorphic information index (PIC; Lu et al., 2005). STRUCTURE 2.3.4 software a model based approach was used for data analysis to obtain possible population structure (Pritchard et al., 2000). We ran STRUCTURE 2.3.4 software with model parameter set of “possibility of admixture and allele frequency correlated” with a burn-in period of the 150,000 followed by 150,000 Markov Chain Monte Carlo (MCMC) replications. Each *K*-value was run for 10 times with *K*-value ranging from 1 to 10. The optimum *K*-value was determined by plotting the log posterior probability data to the given *K*-value. The maximal value of L (K) was identified using the exact number of sub-populations. The model choice criterion to detect the most probable value of K was ΔK, an *ad-hoc* quantity related to the second-order change of the log probability of data with respect to the number of clusters inferred by STRUCTURE (Evanno et al., 2005). Structure Harvester was used for estimation of the Δ*K*-value as function of K showing a clear peak as the optimal *K*-value (Earl and Von, 2012). A total of 60 linked markers distributed on 12 chromosomes were adopted to divide the accessions into different groups with the membership probabilities threshold of 0.80 as well as the maximum membership probability among groups. Those accessions with <0.80 membership probabilities were retained in the admixed group. NEI coefficient for dissimilarity index was calculated by constructing unweighted neighbor joining un-rooted tree (Nei, 1972) with bootstrap value of 1,000 by using DARwin5 software (Perrier and Jacquemoud-Collet, 2006). The Principal Coordinate analysis of the germplasm lines was performed as per the standard published method followed in published paper (Pradhan et al., 2016). GenAlEx 6.5 software was used to assess the presence of molecular variance within and between the population structures using analysis of molecular variance (AMOVA; Peakall and Smouse, 2012). F statistics including deviations from Hardy-Weinberg expectation across the whole population (F_IT_), deviation from Hardy-Weinberg expectation within a population (F_IS_) and correlation of alleles between subpopulation (F_ST_) was calculated. The hypothesis of the association of SSR markers with seedling stage cold tolerance was tested using a general linear model (GLM) and mixed linear model (MLM) in the program TASSEL 5 (Bradbury et al., 2007). Linkage disequilibrium plot was constructed using LD measured r2, between pair of markers is plotted against the distance between the pair. FDR adjusted *p*-values (*q*-values) were estimated using statistical software SPSS version 16.

## Results

### Phenotypic screening of rice genotypes for seedling stage cold tolerance under field situation

**Figure 1:**
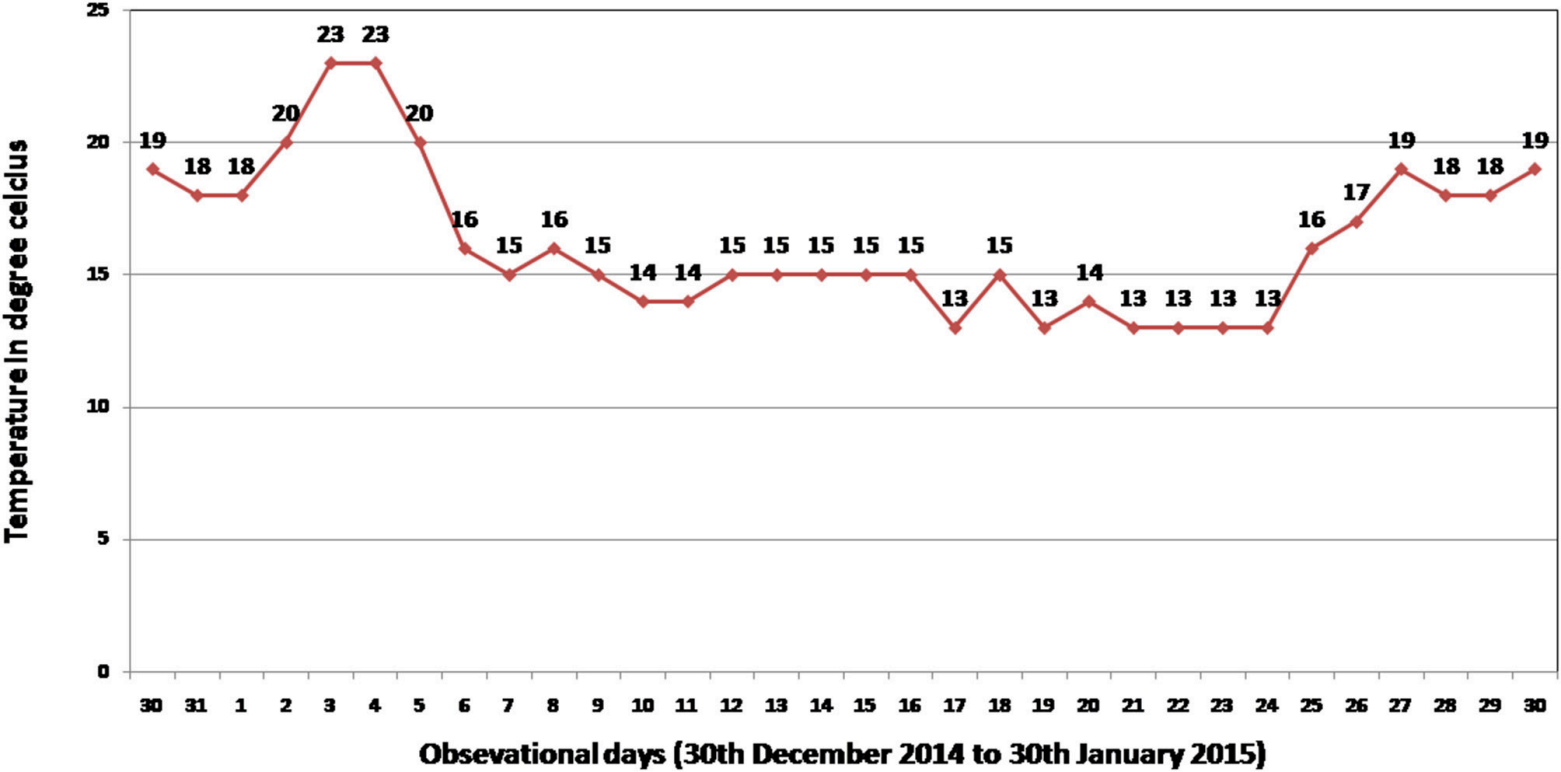
**Minimum temperature recorded at National Rice Research Institute (NRRI), Cuttack, India during field screening period (30th December 2014–30th January in 2015) for seedling stage cold stress tolerance of 304 germplasm lines**.

Phenotypic screening experiment was performed using 304 shortlisted rice germplasm lines during 2014-15 dry season at ICAR-National Rice Research Institute (NRRI), Cuttack, Odisha, India. The shortlisted genotypes were pooled on the basis of its area of adaption in high-altitude area of the country and cold tolerance reaction of the lines from NRRI germplasm catalog. These genotypes were raised during dry season to coincide with the seedling stage of the crop to the coldest period of the year at the Institute site (Figure 1). The field screening results exhibited 86 genotypes to be tolerant to seedling stage cold stress with a score of 1–5. The screening score ranged from SES score of 1 (Geetanjali, KalingaIII, Langma and Umleng-1) to score 9 (Aujari, CR Dhan 505, CR 2340-1, CR 3820-4-5-3-4-1, 9022, Sarpung, Kala Joha, Champa, Hema, Sadabahar, Swarnaprava, Mayang khang, Mayang khang-2, and Yentie) (Table S1). On the basis of subtle changes in leaf color, the shortlisted genotypes for seedling stage cold tolerance under field condition showed 1.3% to be highly tolerant (Score 0–1); 11% tolerant (score 2–3); 16% moderately tolerant (score 4–5); 67% sensitive (score 6–7), and 5% very highly sensitive (score 8–9). A panel population containing 66 genotypes was constituted representing all phenotypic groups for phenotyping under control facility and association mapping of the trait.

### Phenotyping of panel population for seedling stage cold tolerance under control facility

Under field screening, 86 genotypes were found to be tolerant to seedling stage cold with a score of 1–5. Rest, 218 genotypes were observed to be susceptible to cold stress. The screening experiment resulted from control facility by using a cold chamber is presented in Table 1. Under LTR1 exposure, susceptible check variety Sahabhagidhan became brownish yellow with rolled leaf showing SES score of >5 whereas rest 65 genotypes showed normal growth and color. The tolerant genotypes under LTR1 were further exposed to LTR2 to separate out susceptible genotypes in this temperature regime. The 15 separated genotypes were Tejaswini Changlei-1, Manipur local 2, Phouurel, Photem, Manipur local1, Napdai, Sadabahar, Swarnaprava, Swarna, Pratikhya, Tapaswini, Gayatri, Ranidhan, and Naveen showed rolled leaf, yellow to brown in color and reduced growth. Hence, the above two types of susceptible genotypes were categorized into two susceptible sub-groups. Genotypes showing >5 SES score at evaluation temperature of >15°C for 7 days as highly susceptible (HS) types while genotypes exhibiting >5 SES score at above chilling stress (>8°C) and <15°C for 7 days as moderately susceptible (MS) genotypes. The remaining 49 genotypes showing a score of 1–5 with regard to leaf color and leaf rolling at 8°C were further exposed to LTR3, subsequently under which 20 genotypes were observed with score of >5. These 20 genotypes can be grouped as moderately tolerant group to chilling stress at seedling stage (Table 1). Rest 29 genotypes with a score of 1–5 were further exposed to LTR4 (chilling stress up to 14 days at 4°C), only 2 genotypes namely Tabadugu and Krishnahamsa were found to be with SES score of >5 for chilling stress tolerance under this temperature regime. Hence, Tabadugu and Krishnahamsa were categorized as a different group in relation to previous classes and termed as tolerant group. Rest 27 genotypes with 1–5 score were further exposed to 21 days under chilling stress for further grouping under tolerant sub-group (LTR5). At 4°C for 21 days, genotypes exhibiting >5 SES score were Kalinga-3, Radang, Govind, Rungpchi, Ajay, Kamesh, Paun, Manipuridhan, MR37, Umleng-2, Jamak, Charmui, Umbo, Langme-1, Mopu, Tazek, Serum, Bamak, Lagmin, Itanagardhan, and Langme-2. This sub group can be categorized into highly tolerant (HT) group. Genotypes Geetanjali, Langma, and Umleng-1 exhibited a score of 1–5 after 21 days at chilling stress which are categorized as very highly tolerant (VHT) group. The highly tolerant genotypes like Kalinga III (positive check) could survive for 20 days and VHT genotypes AC 43281 (Langma), AC 43291 (Umleng 1) and Geetanjali even survived for 24 days with a score of 7 under exposure to LTR6 chilling stress. Representative figures of different classes of tolerant and susceptible genotypes are depicted in the Figure S1.

### Genetic relatedness by biplot, principal coordinate, and cluster analyses

**Figure 2:**
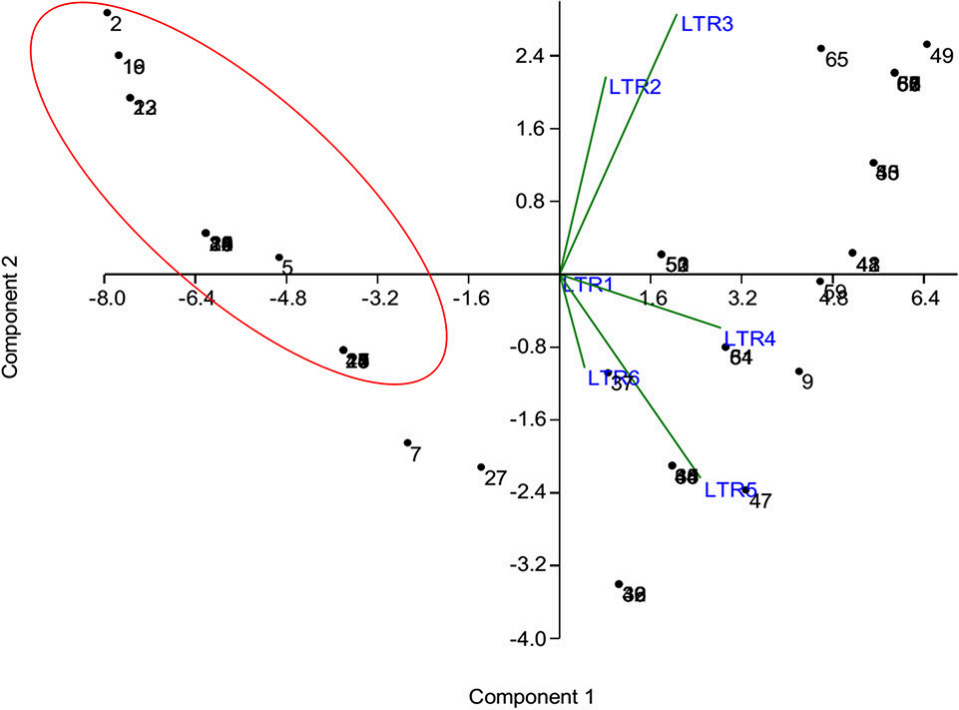
**Genotype-by-trait biplot analysis of 66 genotypes for first two principal components**. LTR1-low temperature regime of 25°C for 3 days; LTR2-low temperature regime of 15°C for 7 days; LTR3-low temperature regime of 8°C for 7 days; LTR4-low temperature regime of 4°C for 7 days; LTR5-low temperature regime of 4°C for 14 days; LTR6-low temperature regime of 4°C for 21 days. The numbers in the figure represent the serial number of the genotypes enlisted in Table 1.

**Figure 3:**
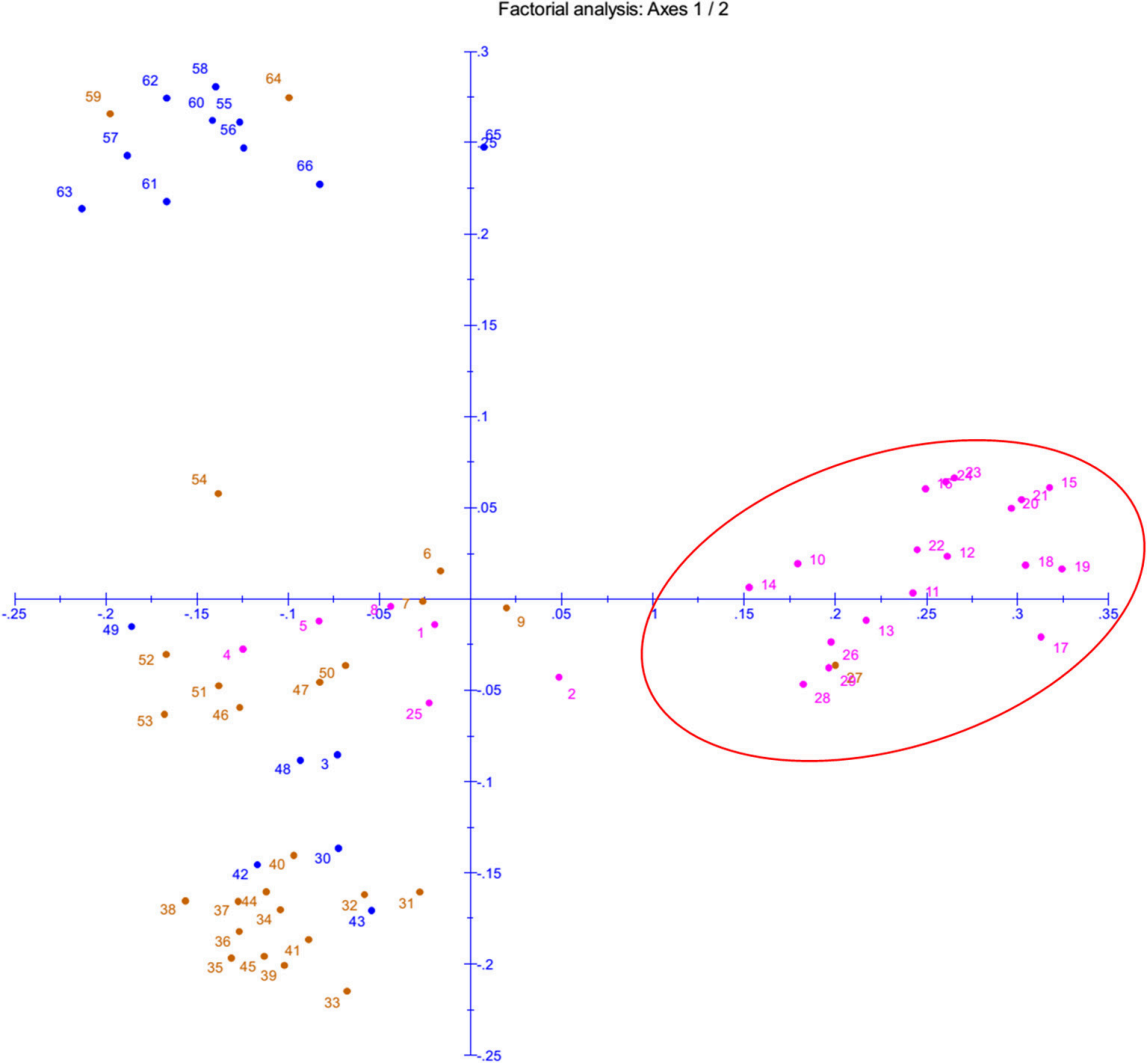
**Principal Coordinate Analysis (PCoA) of panel population based on seedling stage cold tolerance linked 58 SSRs and 2 gene specific markers**. The numbers in the figure represent the serial number of the genotypes enlisted in Table 1.

The phenotyping data of the panel population for chilling stress tolerance were used to produce genotype-by-trait biplot graph for depicting the genotypes in the first two principal components (Figure 2). The first principal component showed 82.51% of variation, while second component explained 11.03% of the total variability. Among the temperature regime studied, LTR3 contributed maximum toward diversity, followed by LTR5 and LTR4 (Figure 2). The top left (Ist quadrant) and bottom left (4th quadrant) accommodated 24 genotypes, which were tolerant to highly tolerant in response to chilling stress during seedling stage. The two tolerant genotypes in 4th quadrant were clearly separated out from the seven highly tolerant genotypes. The 15 genotypes in the 1st quadrant showed better tolerance than the seven highly tolerant genotypes in 4th quadrant. The 2nd quarter contained all the intolerant genotypes to the stress exhibiting higher bronzing symptoms along with few moderately tolerant genotypes. These moderately tolerant genotypes were placed near the axis away from the intolerant ones. The 3rd quadrant genotypes that constituted only moderately tolerant genotypes were marginally better tolerant than the genotypes in 2nd quadrant. The encircled area in the Figure 2 exhibited 22 highly tolerant genotypes. Principal coordinate analysis (PCoA) was performed using the linked markers for determining the genetic relatedness among the genotypes (Figure 3). In PCoA, the desirable genotypes are depicted in the encircled area most of which are located in 1st quadrant. The pattern of distribution of genotypes showed a clear grouping based on tolerance level to chilling stress. The genotypes Umleng-2, Mopu, Umleng-1, Jamak, Manipuri dhan, Tazek, Langma, Paun, MR37 (Farmer selection), Rungpchi, Lagmin, Langme-2, Itanagar dhan, and Tazek clustered together depicted in the encircled area were high to VHT to chilling stress (Figure 3). The first two axis of differentiation explained 19.85 and 14.11% of the total variation, respectively.

**Figure 4:**
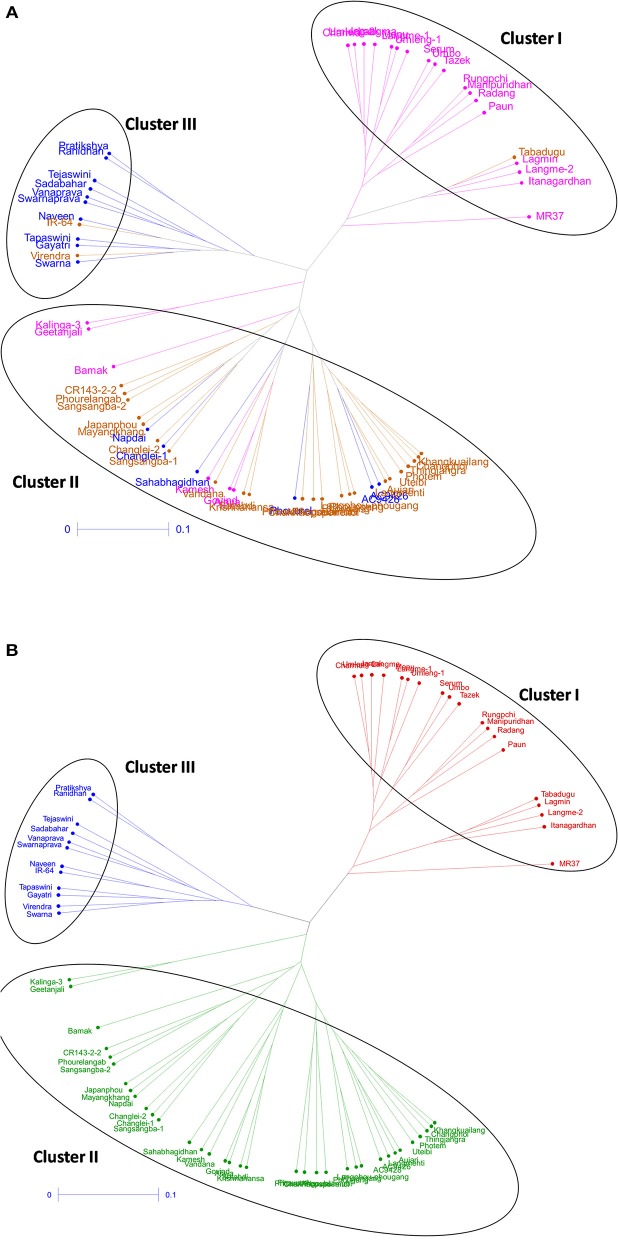
**Unrooted tree using UPGMA method showing the genetic relationship among 66 genotypes based on 60 molecular markers colored on the basis of (A)** individual clusters from origin point and **(B)** sub-populations obtained from structure analysis (SP1-red; SP2-blue, and SP3-green).

Cluster analysis was carried out to assess genetic distance and the dissimilarity matrix-using UPGMA method. The tree constituted of three major clusters (Figure 4). While the phenotyping results of panel population was compared with the grouping pattern, significant correlation was observed (Figure 4A). Cluster 1 possessed 19 genotypes accommodating high to VHT genotypes (pink color) in the tree except Tabadugu (Figure 4A). Similarly, the cluster 2 contained all the sensitive genotypes to chilling stress except IR-64 and Virendra. The moderately tolerant and tolerant germplasm lines were mainly observed in cluster 3 along with few highly tolerant and susceptible types indicating grouping of the admixture type landraces for the trait. However, the UPGMA tree correlated with population structure analysis, the entire genotypes were clearly categorized into three groups and depicted in three colors (Figure 4B). The first cluster consisted all the 19 genotypes belonging to sub-population 1 (red colors). Similarly, cluster 2 (blue color) consisted 12 genotyped classified as SP2 by structure analysis, whereas cluster 3 (green) consisted rest 35 genotypes that were classified as SP3.

### Genetic diversity

The panel containing 66 genotypes, comprising tolerant and susceptible types were genotyped using 58 SSR linked and 2 gene specific markers for the trait. All the loci used for genotyping the panel rice germplasm lines and their genetic diversity parameters obtained are presented in Table 3. The total numbers of amplicons were 222 by using 58 co-dominant and 2 dominant markers. An average of 3.6667 alleles per locus was detected with a range of 1–10 per marker with the highest number of 10 alleles from RM 1812 in the panel for seedling stage chilling stress tolerance. The mean PIC value was observed to be 0.4540 with minimum value of 0.000 (RM200, RM315, RM5221, RM6091, RM6947, and RM 6547) and maximum of 0.7787 (RM1812). The observed average heterozygosity (H_o_) was 0.1912 which ranged between 0.00 and 1.0. It is observed that 42 markers exhibited the level of H_o_ more than zero, while remaining 18 showed zero values. The average heterozygosis or average gene diversity (H_e_) was found to be 0.5069 which varied from 0.0000 (RM319, RM522, RM315, RM200, RM6091, RM6947, and RM6547) to 0.7990 (RM1812). The major allele frequency of these chilling stress tolerance linked polymorphic markers ranged from 0.2879 to 1.0000 with an average of 0.5792 (Table 3).

**Table 3:** **Details of SSR loci used for genotyping a set of 60 rice genotypes and their genetic diversity parameters**.

**Sl. no**.	**Marker**	**No of alleles**	**Minimum size of allele (bp)**	**Maximum size of allele (bp)**	**Major allele frequency**	**Gene diversity**	**Hetero-zygosity**	**PIC value**
1	RM 3375A	4.0000	180	210	0.4318	0.6622	0.0758	0.5974
2	RM 245	4.0000	130	150	0.5455	0.5220	0.0303	0.4145
3	RM 3602	4.0000	140	170	0.3788	0.7256	0.0606	0.6769
4	RM 1347	5.0000	90	150	0.4545	0.6829	0.0758	0.6318
5	RM 5746	5.0000	140	170	0.4167	0.6818	0.0909	0.6214
6	RM 5704	5.0000	160	210	0.3788	0.7084	0.2424	0.6552
7	RM 286	6.0000	100	150	0.4545	0.7285	0.2273	0.6987
8	RM 297	6.0000	150	200	0.3182	0.7694	0.1212	0.7332
9	RM 328	4.0000	180	200	0.4091	0.7103	0.0000	0.6604
10	RM 152	6.0000	70	160	0.3333	0.7480	0.3182	0.7058
11	RM 1341	4.0000	160	190	0.5379	0.6059	0.0758	0.5429
12	RM 84	6.0000	110	150	0.3712	0.7471	0.0455	0.7078
13	RM 472	3.0000	290	330	0.6667	0.4885	0.0000	0.4280
14	RM 85	4.0000	80	110	0.3258	0.7343	0.1212	0.6848
15	RM 341	3.0000	140	180	0.4470	0.6232	0.0152	0.5441
16	RM 561	4.0000	180	200	0.5909	0.5689	0.0000	0.5089
17	RM 493	7.0000	220	300	0.4091	0.7296	0.1667	0.6887
18	RM 7003	3.0000	90	110	0.7879	0.3566	0.0758	0.3283
19	RM 14978	3.0000	200	220	0.4242	0.6538	0.0000	0.5803
20	RM 253	4.0000	120	160	0.4470	0.6190	0.8182	0.5420
21	RM319	1.0000	140	140	1.0000	0.0000	0.0000	0.0000
22	RM 2799	3.0000	130	140	0.5985	0.5031	0.0455	0.4035
23	RM 506	4.0000	120	150	0.4470	0.6467	0.0606	0.5785
24	RM522	1.0000	150	150	1.0000	0.0000	0.0000	0.0000
25	RM315	1.0000	140	140	1.0000	0.0000	0.0000	0.0000
26	RM200	1.0000	150	150	1.0000	0.0000	0.0000	0.0000
27	RM284	3.0000	140	160	0.4091	0.6524	0.0000	0.5778
28	RM 50	4.0000	180	210	0.3409	0.7165	0.0303	0.6634
29	RM 3648	6.0000	170	220	0.3788	0.7319	0.8333	0.6894
30	RM 239	5.0000	100	150	0.4394	0.6125	0.7424	0.5344
31	RM2634	6.0000	65	230	0.4848	0.6964	0.2727	0.6610
32	RM256	4.0000	70	300	0.5152	0.6240	0.4545	0.5591
33	RM305	2.0000	200	210	0.8788	0.2130	0.0000	0.1903
34	RM1211	4.0000	190	240	0.5000	0.5148	0.0303	0.3972
35	RM1812	10.0000	100	295	0.3712	0.7990	0.0758	0.7787
36	RM558	4.0000	130	250	0.6818	0.4927	0.2727	0.4525
37	RM4112	6.0000	130	230	0.5303	0.6149	0.0606	0.5550
38	RM5310	5.0000	140	180	0.5227	0.6258	0.0455	0.5670
39	RM6091	1.0000	120	120	1.0000	0.0000	0.0000	0.0000
40	RM6947	1.0000	150	150	1.0000	0.0000	0.0000	0.0000
41	RM13335	5.0000	140	200	0.3333	0.7246	0.1364	0.6739
42	RM14960	3.0000	110	190	0.5000	0.5147	0.9394	0.3969
43	RM7179	3.0000	120	170	0.6364	0.4830	0.3182	0.3921
44	RM282	2.0000	140	220	0.8409	0.2676	0.3182	0.2318
45	RM6356	3.0000	160	175	0.8636	0.2392	0.0303	0.2169
46	RM3701	5.0000	95	250	0.4394	0.6838	0.4697	0.6288
47	RM26632	2.0000	95	190	0.5000	0.5000	1.0000	0.3750
48	RM173	4.0000	125	500	0.4242	0.6760	0.8030	0.6173
49	RM590	2.0000	140	150	0.8636	0.2355	0.0000	0.2078
50	RM1113	2.0000	155	180	0.5076	0.4999	0.1061	0.3749
51	RM6547	1.0000	160	160	1.0000	0.0000	0.0000	0.0000
52	RM11239	2.0000	400	450	0.8485	0.2571	0.3030	0.2241
53	RM6651	2.0000	140	220	0.8788	0.2130	0.2424	0.1903
54	IN1C3	3.0000	250	280	0.4697	0.6056	0.0000	0.5232
55	IN11D1	2.0000	170	170	0.8485	0.2571	0.0000	0.2241
56	RM9	5.0000	130	220	0.2879	0.7669	0.5758	0.7276
57	RM22491	2.0000	150	155	0.5455	0.4959	0.0000	0.3729
58	RM104	4.0000	160	370	0.3106	0.7416	0.7121	0.6937
59	RM3739	2.0000	130	140	0.5606	0.4927	0.0000	0.3713
60	RM18776	4.0000	150	190	0.8636	0.2475	0.0303	0.2375
	Mean	3.6667			0.5792	0.5069	0.1912	0.4540

### Population structure

**Figure 5:**
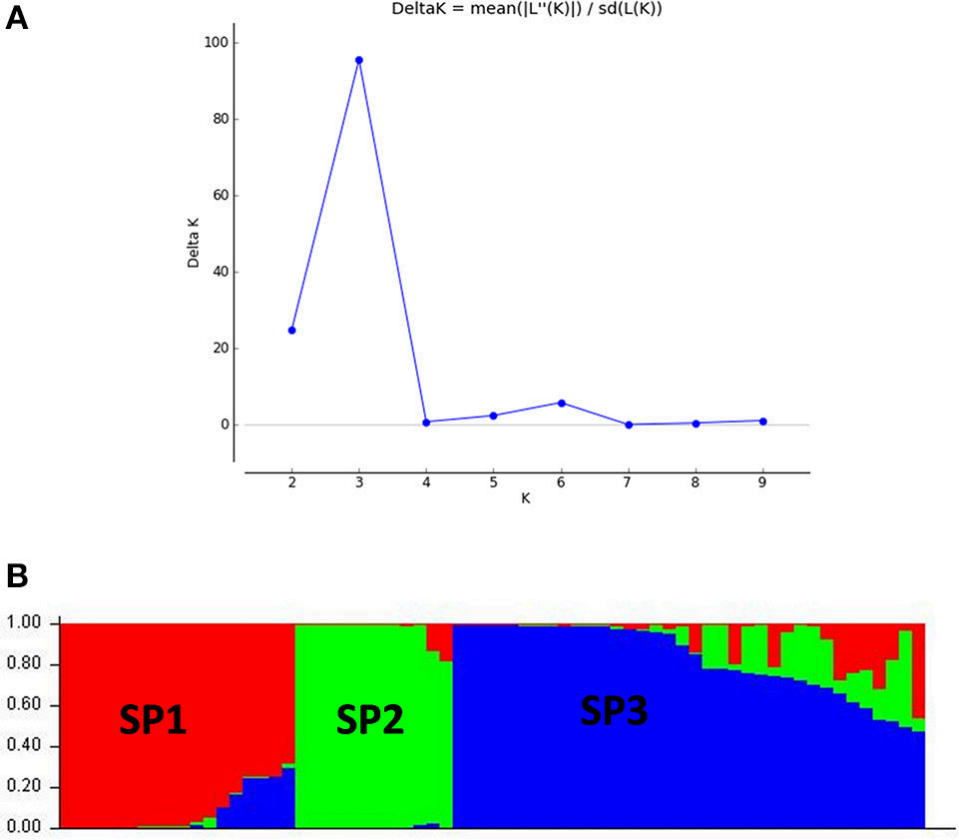
**(A)** Graph of estimated membership probability fraction for *K* = 3. The maximum of *ad-hoc* measure ΔK determined by structure harvester was found to be *K* = 3, which suggested that the entire population can be categorized into three sub-groups (SP1, SP2, and SP3) and **(B)** population structure of a panel possessing 66 genotypes based inferred ancestry using 60 molecular markers (*K* = 3).

**Figure 6:**
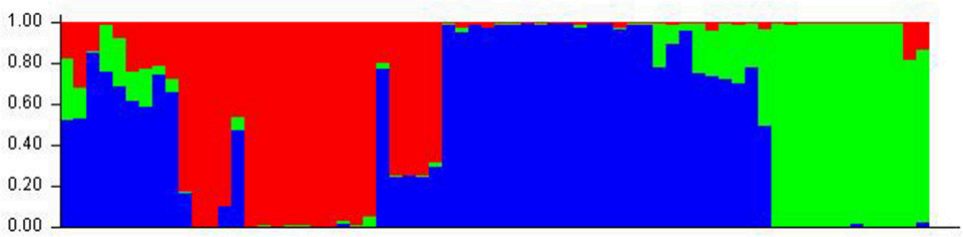
**Population structure of a panel of 66 landraces and breeding lines arranged on the basis of membership probability fractions of individual genotypes**. The membership fractions, the genotypes with the probability of ≥ 80% were assigned to corresponding subgroups with others categorized as admixture.

The panel population was divided into three sub-populations for chilling stress tolerance on the basis of analysis by STRUCTURE software (Figures 5B, 6). The population panel was analyzed for genetic structure on the Bayesian clustering approach taking probable sub-populations (K) and selecting higher Δ*K*-value, an *ad-hoc* quantity related to the second order change of the log probability of data for the number of clusters detected by Structure (Evanno et al., 2005). A high ΔK peak value of 95.6 was observed among the assumed K at *K* = 3 as per the Evano table output (Figure 5A). The sub-population 1 (SP1) contained 18 genotypes with 14 pure and 4 admixture types, accommodating highly and VHT genotypes to seedling stage cold tolerance. A total of 12 genotypes present in sub-population 2 (SP2) representing susceptible sub-population were with all pure type to the sub-population (Figures 5B, 6 and Table 4). The third sub-population can be grouped as moderately tolerant group with 24 tolerant, 6 high to VHT, and 6 susceptible germplasm lines in it. This sub-population has 19 pure and 17 admixture ones genotypes in it. Maximum allele frequency divergence between populations was observed in SP1 and SP2 (0.1664) while within divergence sub population was highest in SP3 (0.242). The fixation index values (F_ST_) of the sub-populations were found to be 0.4947, 0.4663, and 0.3297 for SP1, SP2, and SP3, respectively. Further, the program exhibited a lower value of alpha (α = 0.1079) for the population panel. The distribution pattern of α-value in the panel population and distribution of F_ST_ values in the sub-populations are shown in Supplementary File (Figure S1).

**Table 4:** **Population structure group of accessions based on inferred ancestry values**.

**Sl. no**.	**Genotypes**	**Inferred ancestry**			**Structure group**	**Response to chilling stress tolerance**
		**Q1**	**Q2**	**Q3**		
1	KalingaIII	0.174	0.298	0.528	SP3	HT
2	Geetanjali	0.318	0.152	0.530	SP3	VHT
3	Sahabhagi dhan	0.138	0.011	0.851	SP3	HS
4	Govind	0.012	0.229	0.760	SP3	HT
5	Ajaya	0.078	0.231	0.691	SP3	HT
6	Satabdi	0.239	0.142	0.619	SP3	MT
7	Krishnahans	0.222	0.191	0.587	SP3	T
8	Kamesh	0.208	0.044	0.748	SP3	HT
9	Vandana	0.276	0.062	0.661	SP3	MT
10	Paun	0.825	0.007	0.169	SP1	HT
11	Radang	0.995	0.003	0.002	SP1	HT
12	Manipuridhan	0.996	0.002	0.002	SP1	HT
13	Rungpchi	0.895	0.002	0.102	SP1	HT
14	MR37	0.464	0.061	0.475	SP3	HT
15	Umleng-2	0.996	0.003	0.001	SP1	HT
16	Langma	0.988	0.006	0.005	SP1	VHT
17	Charmui	0.997	0.001	0.001	SP1	HT
18	Jamak	0.990	0.007	0.002	SP1	HT
19	Umleng-1	0.992	0.001	0.006	SP1	VHT
20	Langme-1	0.996	0.002	0.002	SP1	HT
21	Mopu	0.996	0.002	0.001	SP1	HT
22	Tazek	0.970	0.013	0.018	SP1	HT
23	Umbo	0.991	0.005	0.004	SP1	HT
24	Serum	0.944	0.052	0.004	SP1	HT
25	Bamak	0.196	0.031	0.772	SP3	HT
26	Lagmin	0.750	0.004	0.247	SP1	HT
27	Tabadugu	0.746	0.004	0.250	SP1	T
28	Langme-2	0.748	0.005	0.247	SP1	HT
29	Itanagardha	0.682	0.023	0.295	SP1	HT
30	Phouurel	0.004	0.005	0.991	SP3	MS
31	Chakhaopspo	0.026	0.024	0.950	SP3	MT
32	PhourelAngo	0.009	0.002	0.989	SP3	MT
33	Phourelamub	0.026	0.002	0.971	SP3	MT
34	Phougang	0.004	0.007	0.989	SP3	MT
35	Phoungang	0.003	0.011	0.987	SP3	MT
36	Langphou-ph	0.004	0.003	0.993	SP3	MT
37	Changphoi	0.003	0.010	0.988	SP3	MT
38	Uteibi	0.003	0.003	0.994	SP3	MT
39	Khangkuaila	0.002	0.001	0.997	SP3	MT
40	Thingjangra	0.011	0.013	0.976	SP3	MT
41	Photem	0.004	0.002	0.994	SP3	MT
42	AC9426	0.003	0.004	0.993	SP3	MS
43	AC9428	0.027	0.002	0.971	SP3	MS
44	Langmenti	0.003	0.008	0.990	SP3	MT
45	Aujari	0.006	0.003	0.991	SP3	MT
46	Japanphou	0.004	0.217	0.779	SP3	MT
47	Mayangkhang	0.013	0.090	0.897	SP3	MT
48	Napdai	0.005	0.036	0.959	SP3	MS
49	Changlei-1	0.003	0.247	0.751	SP3	MS
50	Changlei-2	0.038	0.220	0.741	SP3	MT
51	Sangsangba-	0.005	0.270	0.726	SP3	MT
52	Phourelanga	0.008	0.285	0.706	SP3	MT
53	Sangsangba-	0.003	0.215	0.782	SP3	MT
54	CR143-2-2	0.035	0.470	0.495	SP3	MT
55	Sadabahar	0.004	0.993	0.003	SP2	MS
56	Tejaswini	0.011	0.983	0.006	SP2	MS
57	Vanaprava	0.002	0.993	0.005	SP2	MS
58	Swarnaprava	0.007	0.989	0.005	SP2	MS
59	Virendra	0.002	0.996	0.002	SP2	MT
60	Swarna	0.003	0.995	0.002	SP2	MS
61	Tapaswini	0.007	0.973	0.020	SP2	MS
62	Gayatri	0.002	0.997	0.002	SP2	MS
63	Naveen	0.002	0.995	0.003	SP2	MS
64	IR-64	0.006	0.993	0.001	SP2	MT
65	Pratikshya	0.182	0.814	0.003	SP2	MS
66	Ranidhan	0.130	0.848	0.023	SP2	MS

*HS, Highly susceptible; MS, Moderately susceptible; MT, Moderately tolerant; T, Tolerant and HT, Highly Tolerant; VHT, Very highly tolerant*.

### Analysis of molecular variance (AMOVA)

The three populations obtained through structure analysis were used for genetic variation between and within the clusters using AMOVA (Table 5). The analysis accounted 30% of the variation among populations, 70% among individuals while no variation detected within individuals in the panel population. Hardy-Weinberg deviation in the population was detected using Wright's F statistic. Similar value of F_IS_ and F_IT_ for all the 60 markers loci were 1.00, while F_ST_ was 0.298 among populations. Pair wise F_ST_ values showed significant differentiation among all the pairs of sub-populations ranging from 0.001 to 0.403 indicating that all the three groups were significantly different from each other. A higher F_ST_ values were observed for pair wise sub-populations in SP1 and SP2 (0.403), SP3 and SP1 (0.281) and Sp3 and SP2 (0.264) when three inter sub-populations are considered. The F_ST_ values and their distribution pattern show clear differentiation of sub populations from each other (Figure S1).

**Table 5:** **Analysis of molecular variance (AMOVA) for the three sub-populations of panel population for seedling stage cold tolerance in rice containing 66 accessions**.

**Source of variation**	**d.f**.	**Mean sum of squares**	**Variance components**	**Percentage variation**
Among populations	2	476.7	10.84	30
Among individuals (accessions) within population	63	51.2	25.58	70
Within individuals (accessions)	66	0.00	0.00	0
Total	131		36.42	100
***F*****-Statistics**	**Value**	***P*****-value**		
F_ST_	0.298	0.001		
F_IS_	1.000	0.001		
F_IT_	1.000	0.001		

### Association of marker alleles with cold tolerance

The marker-trait associations for cold tolerance with various temperature regime and treatment duration were calculated using GLM and MLM (Q+K) model of TASSLE5 software. The comparisons were filtered with *p* < 0.05. The *r*^2^-values varied from 0.0594 to 0.4062 with an average of 0.164 by using GLM, whereas the average reduced to 0.0919 with upper border value of 0.283 and lower border 0.062 by using MLM analysis (**Table 7** and Table S2). Among 60 markers used, 48 markers were associated with different level of cold tolerance by using GLM, whereas the number reduced to 23 with MLM model at *p* < 0.05 and *r*^2^ > 0.05 (Table 6). In total, 130 comparisons with GLM and 8 comparisons with MLM were significant with *r*^2^ > 0.10 at *p* < 0.05. All 130 comparisons significant with GLM at *p* < 0.05 were also significant at *p* < 0.01, but only 5 comparisons were significant with MLM at *p* < 0.05 and *r*^2^ > 0.10.

**Table 6:** **Association of marker alleles with seedling stage cold stress tolerance showing**.

**Seedling stage cold tolerance**	**Marker**	**GLM**				**MLM**			
		***F*****-value**	***P-*****value**	***R*****^2^**	***q*****-value**	***F*****-value**	***P-*****value**	***R*****^2^**	***q-*****value**
7 days at 15°C	RM 328	8.67827	0.00449	0.11941	0.03787	5.33066	0.02419	0.08201	0.05
	RM1812	31.96339	3.93E-07	0.33308	0.007576	6.8187	0.01122	0.1049	0.033333
	RM558	43.77263	8.73E-09	0.40616	0.001515	7.35657	0.00857	0.11318	0.016667
7 days at 8°C	RM 3375A	15.78564	1.83E-04	0.19785	0.010256	4.23706	0.04363	0.06519	0.05
	RM 152	16.38366	1.42E-04	0.20382	0.008974	5.06608	0.02784	0.07794	0.028571
	RM 84	4.28113	0.04258	0.0627	0.048718	5.68895	0.02004	0.08752	0.007143
	RM 472	5.35197	0.02392	0.07717	0.046154	5.18679	0.02611	0.0798	0.021429
	RM2634	30.2678	7.06E-07	0.32108	0.001282	4.35225	0.04095	0.06696	0.042857
	RM1812	11.01544	0.00149	0.14684	0.024359	4.85139	0.03123	0.07464	0.035714
	RM3701	19.97768	3.27E-05	0.23789	0.00641	5.3169	0.02437	0.0818	0.014286
7 days at 4°C	RM284	21.27875	1.96E-05	0.24952	0.011905	4.05799	0.04817	0.06243	0.05
	RM2634	13.43133	5.04E-04	0.17346	0.020238	5.91821	0.01779	0.09105	0.033333
	RM4154	30.64495	6.19E-07	0.32379	0.003571	7.70219	0.00722	0.1185	0.016667
14 days at 4°C	RM 5746	31.32868	4.88E-07	0.32864	0.005	4.55966	0.03657	0.07015	0.042857
	RM 297	10.65265	0.00177	0.1427	0.0275	5.77443	0.01917	0.08884	0.014286
	RM 493	15.18284	2.36E-04	0.19174	0.02125	7.94291	0.00642	0.1222	0.007143
	RM 3648	6.78112	0.01144	0.0958	0.04125	4.9614	0.02944	0.07633	0.028571
	RM 239	6.02904	0.0168	0.08609	0.045	5.45534	0.02265	0.08393	0.021429
	RM5310	18.72681	5.40E-05	0.22637	0.015	4.76148	0.03278	0.07325	0.035714
	RM104	28.07154	1.54E-06	0.30489	0.00875	4.13727	0.0461	0.06365	0.05
21 days at 4°C	RM 1341	30.3578	6.84E-07	0.32173	0.002381	18.41482	6.13E−05	0.2833	0.00625
	RM 493	19.9312	3.33E-05	0.23747	0.004762	6.73344	0.01172	0.10359	0.01875
	RM 7003	6.48717	0.01328	0.09203	0.02381	4.95467	0.02955	0.07623	0.0375
	RM 506	−	−	−	−	4.46335	0.03854	0.06867	0.04375
	RM 50	−	−	−	−	7.51859	0.00791	0.11567	0.0125
	RM2634	−	−	−	−	6.54722	0.01288	0.10073	0.025
	RM1211	5.59002	0.02111	0.08033	0.038095	4.16296	0.04545	0.06405	0.05
	RM5310	4.1527	0.04571	0.06093	0.047619	4.99303	0.02895	0.07682	0.03125

*Significantly higher value in MLM analysis in 66 rice genotypes*.

Cold tolerance at different temperatures like 25, 15, 8, and 4°C at 7, 14, and 21 days treatment were associated with the marker data. Eight markers namely, RM152, RM341, RM50, RM4154, RM245, RM13335, RM282, and RM1341 were associated with cold tolerance at 4°C for 21 days at *p* < 0.01 and *r*^2^ > 0.10 with GLM analysis. Similarly, 28, 30, 34, and 30 numbers of markers were associated with tolerance to 15°C for 7 days, 8°C for 7 days, 4°C for 7 days, and 4°C for 14 days, respectively (Table 6). Higher *F*-value and lower *p*-value with high *r*^2^ indicated the positive association with the trait. Further, MLM analysis was performed to achieve more precise association, considering the kinship value. This showed a strong marker-trait association with phenotypic variance of 11.31% among RM558 and tolerance for 7 days at 15°C to 28.33% among RM1341 and tolerance for 21 days at 4°C considering *p* < 0.01.

**Figure 7:**
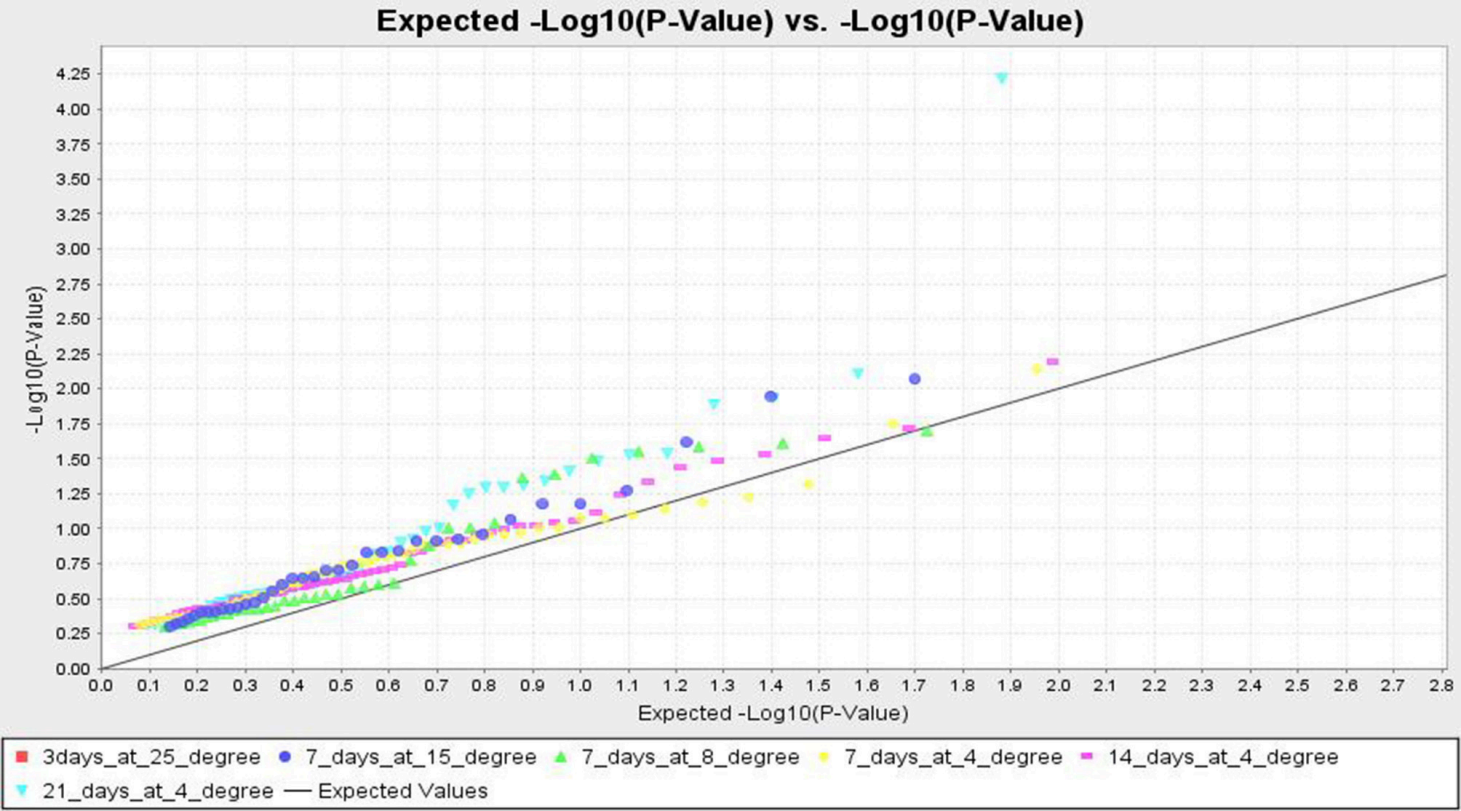
**Quantile–Quantile (QQ) plot and distribution of marker-trait association from MLM analysis at different low temperature regimes**.

TASSEL analysis also evidenced association of some markers with all the studied temperature regimes and duration, whereas some other markers were either regime or duration specific. The markers like RM1347, RM328, RM152, RM341, RM50, RM2634, RM4112, RM5310, RM7179, RM3701, RM104, RM9, and RM1211 were positively associated with all treatments considered, except at 25°C (Table 7 and Table S2). Twelve markers namely, RM3375A, RM5746, RM286, RM84, RM561, RM253, RM284, RM239, RM256, RM1812, RM558, and RM173 were associated with tolerance at 15°C for 7 days to 4°C for 14 days. The markers RM245, RM3602, RM493, RM13335, RM282, and RM5704 did not show any association with tolerance for 7 days at 15°C but all other treatments. Three temperature regime specific markers RM297, RM13341 and RM506 had been detected which were associated with all treatment durations at 4°C, whereas IN11D1 and RM590 were specific for 15°C only. Further, RM6651 and RM22491 were associated with 15°C and 8°C only. The markers IN1C3, RM1113, RM3648 and RM85 were associated with 8°C for 7 days and 4°C for 7 and 14 days but not 21 days. Two markers RM7003 and RM305 showed association with only 21 days treatment at 4°C. However, no significant association was observed for 3 days treatment at 25°C. The QQ plot also confirmed significant association of markers for all temperature regime and treatment duration except 25°C treatment (Figure 7). The linkage disequilibrium decay plot for seedling stage cold tolerance has been plotted using *r*^2^-value between pair of markers and distance between the pair (Figure S2).

**Table 7:** **Association of markers with cold stress at different temperature regimes and durations by using GLM model at *p* < 0.05 and *r*^2^ > 0.05**.

**Sl no**	**Marker name**	**Cold stress treatment**				
		**7 days at 15°C LTR2**	**7 days at 8°C LTR3**	**7 days at 4°C LTR4**	**14 days at 4°C LTR5**	**21 days at 4°C LTR5**
1	RM 1347					
2	RM 328					
3	RM 152					
4	RM 341					
5	RM 50					
6	RM2634					
7	RM4112					
8	RM5310					
9	RM7179					
10	RM3701					
11	RM104					
12	RM9					
13	RM1211					
14	RM 3375A					
15	RM 5746					
16	RM 286					
17	RM 84					
18	RM 561					
19	RM 253					
20	RM284					
21	RM 239					
22	RM256					
23	RM1812					
24	RM558					
25	RM173					
26	RM 14978					
27	RM14960					
28	RM6651					
29	RM22491					
30	RM590					
31	IN11D1					
32	RM 245					
33	RM 3602					
34	RM 493					
35	RM13335					
36	RM282					
37	RM 5704					
38	IN1C3					
39	RM1113					
40	RM 3648					
41	RM 85					
42	RM 472					
43	RM 297					
44	RM 1341					
45	RM 506					
46	RM 2799					
47	RM 7003					
48	RM305					

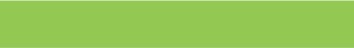
 Associated by using GLM analysis.

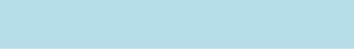
 Not Associated

## Discussion

Low temperature stress during seedling stage is one of the serious yield reducing abiotic factors in dry season rice, particularly boro rice of India and cultivation in the high altitude areas like hill rice. The effect of seedling stage cold prolongs the growth duration of the rice plant, subsequently delay in flowering period of boro and dry season rice that coincides with high temperature stress period resulting in drastic reduction in yield (Pradhan et al., 2016). Therefore, cold tolerance breeding for seedling stage is important in boro and dry season rice. Results of field screening experiment followed by controlled screening method under RGA-cum-Phytotron, we could identify 50 germplasm lines with seedling stage cold tolerance. The genotypes were classified into six classes based on their tolerance to cold stress after exposure to six temperature regimes. Clustering by using results on molecular markers and genotype-trait biplot analysis exhibited grouping of genotypes basing on their tolerance to seedling stage cold tolerance (Figures 2–4). The tolerant genotypes were grouped into many sub-groups depending upon their level of tolerance, which might be the result of expression of different gene(s)/QTL(s) for seedling stage cold tolerance and their possible presence in the studied materials. Hence, the population structure of the panel population for the trait is most important. Earlier studies also indicated screening and identification of cold tolerant genotypes in rice (Kwak et al., 1984; Nagamine, 1991; Kim et al., 2000, 2014; Misawa et al., 2000; Qian et al., 2000; Andaya and Mackill, 2003; Qu et al., 2003; Fujino et al., 2004; Zhan et al., 2005; Zhang et al., 2005; Andaya and Tai, 2006; Jiang et al., 2006, 2008; Han et al., 2007; Lou et al., 2007; Koseki et al., 2010; Wang et al., 2011; Suh et al., 2012; Pradhan et al., 2015). Germplasm lines like AC 43281 (Langma), AC 43291 (Umleng 1), and Geetanjali were observed to be VHT to seedling stage cold tolerance. These results are also confirming the earlier screening results for chilling stress tolerance in rice (Pradhan et al., 2015).

The principal component and coordinate analysis placed the tolerant and non-tolerant genotypes into various spots in the four quadrants. The location of the genotypes with respect to origin provides clue for tolerance to seedling stage cold tolerance. Thus, distribution of genotypes in different quadrant confirmed the presence of variation for the trait. The UPGMA tree, categorized the highly tolerant and tolerant classes on the basis of banding pattern of 60 cold stress linked molecular markers. The genotypes could be categorized into separate clusters which are in line with the seedling stage cold tolerance phenotype groups. Various groups and sub-groups of the genotypes obtained based on banding pattern of cold tolerance linked markers suggested the presence of many genes/QTLs in the panel population. In our study, a moderate level of genetic diversity was detected for seedling stage cold tolerance. Our results on the level of genetic diversity for the trait is similar to the other results of moderate diversity parameters reported earlier for various traits (Agrama and Eizenga, 2008; Jin et al., 2010; Chen et al., 2011; Zhang et al., 2011; Shah et al., 2013; Singh et al., 2013). However, very rich genetic diversity values for agro-morphologic traits were also reported earlier in rice (Garris et al., 2003; Zhao et al., 2013; Salgotra et al., 2015).

We demonstrated the appropriateness of the suggested panel population for association mapping and kinship study basing on population structure and relatedness for seedling stage cold tolerance. The phenotyping and genotyping of the panel population using 60 linked markers for the trait clearly categorized the study materials into different groups, suggesting their differential response to cold stress tolerance (Figures 2–4). This heterogeneity favored the presence of linkage disequilibrium and increased the chance of recovering marker-trait association. Marker-phenotypic trait association was also detected earlier showing the potential value of germplasm in heterogeneous collections (Gebhardt et al., 2004; Lu et al., 2005; Caicedo et al., 2007; Zhang et al., 2009, 2011; Zhao et al., 2013; Anandan et al., 2016; Pradhan et al., 2016). Boro and dry season rice affected by low temperature stress during seedling need attention for incorporating seedling stage cold tolerance. The application of association mapping results for this trait will be helpful in selecting the molecular markers to be used in marker-assisted breeding of the multiple QTLs/genes responsible for the trait. Therefore, different landraces with differential response to the stress are needed for association mapping of the trait. In our results, we detected a lower value of alpha (α = 0.1079) from which we can infer that in most of the landraces, the trait had a common primary ancestor with few admix individuals in each sub-population. The inferred ancestry indicated that small effects QTLs for the trait present in different landraces might be pooled together from long time ago through intercrossing of many landraces naturally as a result of which few landraces possess many QTLs that are strongly tolerant to the stress. Similar views have also been provided by previous reports (Mather et al., 2004; Zhao et al., 2013; Pradhan et al., 2016).

Values of F_ST_ were very high in SP1 and SP2 (0.403), SP3 and SP1 (0.281) and SP3 and SP2 (0.264) when combination of inter sub-populations are taken together, thus suggesting higher genetic differences between germplasm accessions. It has been demonstrated that populations and individuals with higher F_*ST*_ values produce better variable materials when combined with lines from different genetic diversity estimates (Watkins et al., 2003). The significant F_ST_ among the clusters indicate a real variation in these clusters, and attempt to pyramid the QTLs governing the trait may improve further tolerance for seedling stage cold tolerance. Similar suggestion was also provided by earlier workers for increasing heterosis for grain yield in rice (N'Goran et al., 2000).

Cold stress exposure at different temperature regimes of the tested panel genotypes was observed to be associated with the marker data. Higher F and lower *p*-value with high *r*^2^ were detected for 28, 30, 34, 30, and 8 markers associated with tolerance to LTR 2 (15°C for 7 days), LTR 3 (8°C for 7 days), LTR 4 (4°C for 7 days), LTR 5 (4°C for 14 days), and LTR 6 (4°C for 21 days), respectively. This indicated more numbers of markers detected by TASSEL using both GLM and MLM analyses providing a robust marker–phenotype association in the present study which was also evident from Q-Q plot (Figure 7). This also suggests that, the observed marker-trait association resulted possibly from multiple introgressions or accumulation of QTLs from the landraces by natural hybridization over a long time period ultimately exhibiting a higher level of tolerance. Similar results on multiple introgressions of tolerant QTLs for high temperature stress from the landraces were also earlier described in rice (Pradhan et al., 2016). In this study, we found a strong marker-trait association by both MLM and GLM models of TASSEL analysis in all the studied temperature regimes with some regime or duration specific markers association except LTR1. This confirms the effectiveness of the linked markers for cold tolerance not showing any significant association with temperature treatment at 25°C. Twelve markers namely, RM3375A, RM5746, RM286, RM84, RM561, RM253, RM284, RM239, RM256, RM1812, RM558, and RM173 were associated with tolerance at 15°C for 7 days to 4°C for 14 days. Tolerance in these temperature regimes suggests that the markers associated at these low temperature exposures were with QTLs/genes conferring tolerance from mild to highly tolerance response to the stress. The phenotype groups associated for these primers were MS, MT, and T types of phenotypes. Similarly, markers like RM1347, RM328, RM152, RM341, RM50, RM2634, RM4112, RM5310, RM7179, RM3701, RM104, RM9, and RM1211 were positively associated with all treatments considered except 25°C. This suggests that tolerance to all the temperature regimes means germplasm lines possessing many tolerance conferring QTLs for cold tolerance. Here, these primers are associated with four phenotype groups observed for the trait. This is also evidenced from the earlier bi-parental mapping population indicating role of QTLs like qCTS11.1, qCTS9, qCTS6-1, qSCT2, qSCT1a, qCTS-3.1, qCTS-2, qCTS12.1, and qCTS-1b for seedling stage cold tolerance (Andaya and Mackill, 2003; Long-zhi et al., 2004; Lou et al., 2007; Wang et al., 2011; Kim et al., 2014). Similarly, markers RM245, RM3602, RM493, RM13335, RM282, and RM5704 did not show any association with tolerance for 7 days at 15°C but all other treatments. This indicates the association of tolerant phenotypes excluding the chilling sensitive alleles. These associations of primers with tolerance phenotypes indicate that the tolerant genotypes chosen here were from all tolerant groups and may possess many tolerant QTLs for the trait. Similar results on high temperature stress tolerance governed by multiple tolerant QTLs were reported earlier in rice (Pradhan et al., 2016). Previous research results on cold stress which suggested presence of many cold tolerant QTLs on chromosome 1, 2, 3, 4, 5, 6, 8, 9, 10, 11, and 12 obtained by using various mapping populations (Andaya and Mackill, 2003; Long-zhi et al., 2004; Lou et al., 2007; Wang et al., 2011; Suh et al., 2012; Kim et al., 2014; Bonnecarrere et al., 2015; Pradhan et al., 2015). Also, our PCA, PCoA, and UPGMA trees revealed presence of highly tolerant lines in the encircled area (Figures 2–4). The study showed that out of the earlier reported QTLs (Table 1), only nine QTLs namely, qCTS11.1, qCTS9, qCTS6-1, qSCT2, qSCT1a, qCTS-3.1, qCTS-2, qCTS12.1, and qCTS-1b were significantly involved for broad level of cold tolerance at seedling stage, whereas the other QTLs were less effective. This may be due to the linked markers chosen for our study which were earlier reported for various related traits through bi-parental mapping approach. When the association mapping was studied in multiple genotypes, it revealed robustness of these QTLs and linked markers suggesting their further use in marker-assisted seedling stage cold tolerance breeding program.

## Conclusion

From the experiment, a moderate level of genetic diversity for seedling stage chilling tolerance was noticed by using the panel population. Results of the STRUCTURE analysis revealed that the entire population could be grouped into three sub-populations and further detected that most of the landraces had a common primary ancestor with few admix individuals for the trait. The donor lines in the panel exhibited the presence of different QTLs representing like whole genome diversity for the expression of tolerance. The significantly associated markers like RM1347, RM328, RM152, RM341, RM50, RM2634, RM4112, RM5310, RM7179, RM3701, RM104, RM9, RM1211, RM245, RM3602, RM493, RM1335, RM282, and RM5704 were significantly associated at chilling stress of 8°C to 4°C for 7–21 days duration. Thus, the primers linked to the seedling stage cold tolerance QTLs namely qCTS9, qCTS-2, qCTS6.1, qSCT2, qSCT11, qSCT1a, qCTS-3.1, qCTS11.1, qCTS12.1, qCTS-1b, and CTB2 need to be pyramided for development of strongly chilling tolerant variety.

## Author contributions

Conceived and designed the experiments: SP, EP. Performed the phenotyping experiments: DN, SD, SM, SP. Performed the genotyping experiments: ST, DN, SB, DM, SD, EP. Analyzed the data: EP. Contributed reagents/materials/analysis tools: SP. Wrote the paper: SP, EP.

### Conflict of interest statement

The authors declare that the research was conducted in the absence of any commercial or financial relationships that could be construed as a potential conflict of interest.
