# Supplementary material for: Genome-Wide Association Mapping Reveals Multiple QTLs Governing Tolerance Response for Seedling Stage Chilling Stress in Indica Rice
Source: Front Plant Sci. 2017 Apr 25;8:552. doi: 10.3389/fpls.2017.00552 (PMC5404645; doi:10.3389/fpls.2017.00552)
Supplement: Supplementary file 1 [file Table1.DOC]

**Table S1** Germplasm lines used in field screening for seedling stage cold tolerance and their SES score under field screening.

| **SL.**  **No.** | **Germplasm**  **Name** | **Accession**  **Number** | **Score** | **Place of collection/**  **adaption** | **SL.**  **No.** | **Germplasm**  **Name** | **Accession**  **Number** | **Score** | **Place of collection/**  **adaption** |
| --- | --- | --- | --- | --- | --- | --- | --- | --- | --- |
| 1 | Phouurel | 9005 | 7(63.2) | Manipur | 153 | Sri Lanka | 43091 | 7(76.2) | Assam |
| 2 | Govind | Variety | 5(90.2) | Uttar Pradesh | 154 | Halli Dhan | 43092 | 7(78.2) | Assam |
| 3 | Aujari | 9006 | 7(80.2) | Manipur | 155 | Maglang | 43093 | 7(87.2) | Assam |
| 4 | Chakhaopspoireitol | 9008 | 5(74.2) | Manipur | 156 | Bora | 43094 | 3(85.2) | Assam |
| 5 | Phourel Angoubi | 9011 | 3(73.2) | Manipur | 157 | Kumari | 43095 | 7(83.2) | Assam |
| 6 | Panikeokia | Cultivar | 7(80.2) | Assam | 158 | Lungbo | 43096 | 7(81.2) | Assam |
| 7 | Phourelamubi | 9200 | 3(78.2) | Manipur | 159 | Phourel-1 | 9024 | 7(83.2) | Manipur |
| 8 | IRRI-120 | Hybrid | 3(82.2) | Philippines | 160 | Lal Aijung | 43097 | 7(87.2) | Assam |
| 9 | Yentie | 9027 | 9(88.2) | Manipur | 161 | Kumbi | 9025 | 7(77.2) | Manipur |
| 10 | Karta dhan | 43268 | 7(64.2) | Arunachal Pradesh | 162 | Phougang | 9201 | 3(73.2) | Manipur |
| 11 | Phoungang | 9216 | 3(66.2) | Manipur | 163 | Laxman | 43098 | 7(79.2) | Assam |
| 12 | Improved Lalat | Variety | 5(66.2) | Odisha | 164 | Mayang khang-2 | 9028 | 9(89.2) | Manipur |
| 13 | CR-2840-1 | Culture | 7(68.2) | CRRI,Cuttack | 165 | Shanti joha | 43099 | 7(77.2) | Assam |
| 14 | Langphou phougang | 9235 | 3(88.2) | Manipur | 166 | Changli | 9029 | 7(87.2) | Manipur |
| 15 | Parijat | Variety | 7(60.2) | Odisha | 167 | Nashket Bora | 43100 | 7(79.2) | Assam |
| 16 | IRRI-143 | Culture | 7(70.2) | IRRI,Philippines | 168 | Moiranghouanganba | 9030 | 5(83.2) | Manipur |
| 17 | Changphoi | 9243 | 3(63.2) | Manipur | 169 | Kunkuni joha | 43102 | 7(77.2) | Assam |
| 18 | CR2837-1 | Culture | 7(78.2) | CRRI,Cuttack | 170 | ketoki joha | 43103 | 7(77.2) | Assam |
| 19 | Ajaya | Culture | 7(82.2) | IRRI,Philippines | 171 | Changli | 9039 | 7(83.2) | Manipur |
| 20 | Uteibi | 9402 | 3(70.2) |  | 172 | Joha Bora | 43104 | 7(71.2) | Assam |
| 21 | Rajalaxmi | Hybrid | 5(88.2) | Odisha | 173 | Ranga Bora | 43105 | 7(79.2) | Assam |
| 22 | Lalat | Variety | 5(72.2) | Odisha | 174 | Phoudum | 9043 | 7(83.2) | Manipur |
| 23 | Annada (check) | Variety | 4(85.2) | Odisha | 175 | Bor-janghia | 43106 | 7(73.2) | Assam |
| 24 | Khangkuailang | 9403 | 3(88.2) | Manipur | 176 | Basumati | 43107 | 7(69.2) | Arunachal Pradesh |
| 25 | Satabdi | Variety | 3(84.2) | West Bengal | 177 | Sangsangba | 9056 | 7(79.2) | Manipur |
| 26 | IRRI-112 | Culture | 7(74.2) | IRRI,Philippines | 178 | Shillong Andeng | 43108 | 5(79.2) | Arunachal Pradesh |
| 27 | Sahabhagidhan (check) | Variety | 7.5(91.5) | Odisha,Jharkhand | 179 | Amro(Dalmang) | 43109 | 7(73.2) | Arunachal Pradesh |
| 28 | Geetanjali | Variety | 1(92.2) | Odisha | 180 | Umkel | 43110 | 7(67.2) | Arunachal Pradesh |
| 29 | Vandana (check) | Variety | 5(85.2) | Jharkhand | 181 | Phourel Khongau | 9057 | 7(83.2) | Manipur |
| 30 | Aujari | 9006 | 9(88.2) | Manipur | 182 | Shillong | 43111 | 7(71.2) | Arunachal Pradesh |
| 31 | Brown gora | 17008 | 7(74.2) | Bihar | 183 | Miching | 43112 | 7(79.2) | Arunachal Pradesh |
| 32 | Chandrama | Variety | 5(60.2) | Assam | 184 | Amdeng | 43113 | 5(73.2) | Arunachal Pradesh |
| 33 | Thingjangra | 9413 | 3(70.2) | Manipur | 185 | Tagur | 43115 | 7(83.2) | Arunachal Pradesh |
| 34 | IRRI-43-s-b | Culture | 7(84.2) | IRRI,Philippines | 186 | Itanagar | 43116 | 7(73.2) | Arunachal Pradesh |
| 35 | Kalinga-III(Check) | Variety | 1(92.8) | Odisha | 187 | Moitangphon | 9066 | 7(83.2) | Manipur |
| 36 | BR 72 | Variety | 7(64.2) | Jharkhand | 188 | Kala Bora | 43117 | 7(79.2) | Assam |
| 37 | CR-2837-1-1-1-2 | Culture | 7(82.2) | CRRI,Cuttack | 189 | Beckjer | 43118 | 7(77.2) | Assam |
| 38 | Photem | 9417 | 7(84.2) | Manipur | 190 | Tumaiphougak | 9080 | 7(87.2) | Manipur |
| 39 | Padmini | Variety | 5(76.2) | Odisha | 191 | Geko | 43119 | 5(79.2) | Assam |
| 40 | Kalakeri | 23652 | 7(62.2) | Odisha | 192 | Darim | 43120 | 5(73.2) | Assam |
| 41 | Manipur Local-1 | 9426 | 7(90.2) | Manipur | 193 | Chhota jangia | 43121 | 7(81.2) | Assam |
| 42 | CR 2839-1-17-1-4 | Culture | 7(84.2) | CRRI,Cuttack | 194 | Kokua | 43122 | 5(79.2) | Assam |
| 43 | CR 1980-1 | Culture | 7(74.2) | CRRI,Cuttack | 195 | Panghan | 9116 | 5(77.2) | Manipur |
| 44 | IRRI 113 | Culture | 7(82.2) |  | 196 | Chokua | 43123 | 7(83.2) | Assam |
| 45 | Virendra | Variety | 7(78.2) | Bangladesh | 197 | Bada jangia | 43124 | 7(77.2) | Assam |
| 46 | Manipur Local-2 | 9428 | 7(88.2) | Manipur | 198 | Ritengdaimai | 9117 | 7(81.2) | Manipur |
| 47 | Pucho | 43239 | 5(76.2) | Arunachal Pradesh | 199 | Alpana | 43125 | 7(75.2) | Assam |
| 48 | Amte | 43240 | 7(86.2) | Arunachal Pradesh | 200 | Gamiri | 43126 | 7(79.2) | Assam |
| 49 | IET 21625 | culture | 7(88.2) | CRRI,Cuttack | 201 | Ampi | 43127 | 5(83.2) | Assam |
| 50 | Damchak | 43242 | 7(80.2) | Arunachal Pradesh | 202 | Joha | 43128 | 5(83.2) | Assam |
| 51 | Langmenti | 9004 | 5(76.2) | Manipur | 203 | Napchong | 9119 | 9(89.2) | Manipur |
| 52 | CR3825-2-1-2-3 | Culture | 7(82.2) | CRRI,Cuttack | 204 | Effcey | 43129 | 7(75.2) | Assam |
| 53 | Kilong | 43244 | 7(84.2) | Arunachal Pradesh | 205 | Bogadhan | 43130 | 7(70.2) | Assam |
| 54 | Adi | 43245 | 7(76.2) | Arunachal Pradesh | 206 | Lahi joha | 43131 | 7(80.2) | Arunachal Pradesh |
| 55 | CRDhan 505 | Variety | 9(93.2) | Assam,Odisha | 207 | Airi | 43132 | 7(76.2) | Arunachal Pradesh |
| 56 | Aujari | 9032 | 3(73.2) | Manipur | 208 | Satyakrishna | Variety | 7(80.2) | Odisha |
| 57 | CR2707 | Culture | 7(85.2) | CRRI,Cuttack | 209 | Ampaki | 43133 | 7(76.2) | Arunachal Pradesh |
| 58 | CR3819-8-1-1-3-1 | Culture | 7(83.2) | CRRI,Cuttack | 210 | Jangia | 43134 | 5(80.2) | Arunachal Pradesh |
| 59 | Japan phou | 9036 | 3(79.2) | Manipur | 211 | Boradhan | 43135 | 5(74.2) | Arunachal Pradesh |
| 60 | Nishin | 43249 | 7(89.2) | Arunachal Pradesh | 212 | Hallidhan | 43136 | 7(72.2) | Arunachal Pradesh |
| 61 | Nagaum | 43250 | 5(85.2) | Arunachal Pradesh | 213 | Jaya | Variety | 5(70.2) | Assam |
| 62 | CR3622-7-3-1-1-1 | Culture | 7(87.2) | CRRI,Cuttack | 214 | Gadra | 43137 | 7(84.2) | Arunachal Pradesh |
| 63 | Mayang khang-1 | 9038 | 5(85.2) | Manipur | 215 | Nania | 43138 | 7(76.2) | Arunachal Pradesh |
| 64 | CR3628-8-3-2-1-1 | Culture | 7(81.2) | CRRI,Cuttack | 216 | Phourel-2 | 9185 | 5(78.2) | Manipur |
| 65 | Napdai | 9153 | 7(91.2) | Manipur | 217 | Aijung | 43139 | 7(86.2) | Assam |
| 66 | Radang | 43248 | 3(89.2) | Arunachal Pradesh | 218 | Birohi | 43140 | 7(84.2) | Assam |
| 67 | Ningpung | 43254 | 3(95.2) | Arunachal Pradesh | 219 | Nagahalli | 43141s | 7(88.2) | Assam |
| 68 | CR3621-6-1-3-1-1 | Culture | 7(87.2) | CRRI,Cuttack | 220 | IR36 | Variety | 5(80.2) | Assam |
| 69 | Paun | 43256 | 5(73.2) | Arunachal Pradesh | 221 | Boro-4004 | Culture | 7(74.2) | CRRI,Cuttack |
| 70 | Bibililo | 43262 | 7(85.2) | Arunachal Pradesh | 222 | Moirangphou | 9191 | 5(76.2) | Manipur |
| 71 | Changlei-1 | 9197 | 7(89.2) | Manipur | 223 | Anjali | Variety | 7(84.2) | Jharkhand |
| 72 | Teze | 43258 | 3(85.2) | Arunachal Pradesh | 224 | Hazaridhan | variety | 7(82.2) | Jharkhand |
| 73 | Changlei-2 | 9204 | 5(71.2) | Manipur | 225 | CRHR-32 | Hybrid | 7(76.2) | Odisha |
| 74 | Rungpchi | 43259 | 3(87.2) | Arunachal Pradesh | 226 | Heera | Variety | 7(72.2) | Odisha |
| 75 | Saap | 43260 | 7(89.2) | Arunachal Pradesh | 227 | N-22 | Landrace | 7(76.2) | Uttar Pradesh |
| 76 | Sangsangba-1 | 9221 | 5(79.2) | Manipur | 228 | Phorel | 9203 | 7(74.2) | Manipur |
| 77 | Tazek | 43261 | 3(93.2) | Arunachal Pradesh | 229 | Jalmagna | Variety | 7(84.2) | Uttar Pradesh |
| 78 | CR3825-6-4-6-1-1 | Culture | 7(95.2) | CRRI,Cuttack | 230 | Phoungang | 9205 | 7(84.2) | Manipur |
| 79 | Phourel angaba | 9227 | 5(73.2) | Manipur | 231 | Satyabhama | Variety | 7(80.2) | Odisha |
| 80 | Itanagar dhan | 43263 | 3(91.2) | Arunachal Pradesh | 232 | Tapaswini | Variety | 7(82.2) | Odisha |
| 81 | CR2340-1 | Culture | 9(91.2) |  | 233 | Moirang | 9212 | 7(82.2) | Manipur |
| 82 | Karta pachung | 43265 | 7(89.2) | Arunachal Pradesh | 234 | Ratna | Variety | 7(80.2) | Odisha |
| 83 | Sangsangba-2 | 9258 | 5(73.2) | Manipur | 235 | Savitri | Variety | 7(84.2) | Odisha |
| 84 | Nepali dhan | 43266 | 7(79.2) | Arunachal Pradesh | 236 | Tumai | 9218 | 7(80.2) | Manipur |
| 85 |  | CR143-2-2 | 5(79.2) | Odisha | 237 | Pooja | Variety | 7(78.2) | Odisha |
| 86 | Naga dhan | 43267 | 7(89.2) | Arunachal Pradesh | 238 | Boro 4006 | Culture | 7(80.2) | CRRI,Cuttack |
| 87 | Sadabahar | variety | 7(83.2) | Jharkhand | 239 | Satyakrishna | Variety | 7(84.2) | Odisha |
| 88 | IRRI-140 | Culture | 7(89.2) | IRRI,Philippines | 240 | Phoudum | 9220 | 7(78.2) | Manipur |
| 89 | Ningte | 43269 | 7(77.2) | Arunachal Pradesh | 241 | Dhala Heera | Variety | 7(72.2) | Odisha |
| 90 | Tejeswini | variety | 7(89.2) | Odisha | 242 | Phourel phondum | 9231 | 7(80.2) | Manipur |
| 91 | Kamesh | Variety | 3(93.2) | Jharkhand | 243 | Abhisek | Variety | 7(86.2) | Jharkhand |
| 92 | Swarnaprava | Variety | 7(83.2) | Andhra Pradesh | 244 | Phourel-3 | 9232 | 5(74.2) | Manipur |
| 93 | Ningche | 43270 | 7(81.2) | Arunachal Pradesh | 245 | Samlei | Variety | 7(88.2) | Odisha |
| 94 | Bombi | 43271 | 3(93.2) | Arunachal Pradesh | 246 | Lunisree | Variety | 7(76.2) | Odisha |
| 95 | Virendra | Variety | 5(89.2) | Jharkhand | 247 | Tumia angonba | 9238 | 9(90.2) | Manipur |
| 96 | Rimi | 43272 | 7(73.2) | Arunachal Pradesh | 248 | Neela | Variety | 7(84.2) | Odisha |
| 97 | Phourel Angoubi | 9010 | 7(85.2) | Manipur | 249 | Aujari | 9248 | 7(84.2) | Manipur |
| 98 | Jijiko (scented) | 43273 | 5(81.2) | Arunachal Pradesh | 250 | Pathara | Variety | 7(76.2) | Odisha |
| 99 | Krishnahansa | Variety | 5(83.2) | Andhra Pradesh | 251 | CR2340-2 | Culture | 7(86.2) | CRRI,Cuttack |
| 100 | Phurel | 9217 | 5(89.2) | Manipur | 252 | Kumbi phou | 9259 | 7(84.2) | Manipur |
| 101 | MR 37 | 43275 | 3(97.2) | Arunachal Pradesh | 253 | CR 2340-11 | Culture | 7(76.2) | CRRI,Cuttack |
| 102 | Mapung | 43276 | 7(85.2) | Arunachal Pradesh | 254 | Phourel Yenthic | 9264 | 7(80.2) | Manipur |
| 103 | Koimurali | 9228 | 5(89.2) | Manipur | 255 | CR2340-1 | Culture | 7(83.2) | CRRI,Cuttack |
| 104 | Kabang | 43277 | 7(83.2) | Arunachal Pradesh | 256 | CR2301-5 | Variety | 7(83.2) | Odisha,Bihar |
| 105 | Legmin | 43278 | 3(90.2) | Arunachal Pradesh | 257 | Phondengba | 9265 | 7(79.2) | Manipur |
| 106 | Moirangphou | 9230 | 7(78.2) | Manipur | 258 | Rudra | Variety | 7(79.2) | Odisha |
| 107 | Naar | 43279 | 7(88.2) | Arunachal Pradesh | 259 | Sankar | Variety | 7(79.2) | Odisha |
| 108 | Manipuri dhan | 43280 | 3(82.2) | Arunachal Pradesh | 260 | IRRI-116 | Culture | 7(81.2) | IRRI,Philippines |
| 109 | Anjari | 9237 | 5(72.2) | Manipur | 261 | Hue | Variety | 7(77.2) | Odisha |
| 110 | Langma | 43281 | 1(92.2) | Arunachal Pradesh | 262 | Kalyani II | Variety | 7(83.2) | Odisha |
| 111 | Iroiya | 9240 | 5(88.2) | Manipur | 263 | Gouri | Variety | 7(81.2) | Odisha |
| 112 | Serum | 43282 | 3(84.2) | Arunachal Pradesh | 264 | Kedar | Variety | 7(81.2) | Odisha |
| 113 | Sarpung | 43283 | 7(72.2) | Arunachal Pradesh | 265 | Vanaprava | Variety | 7(67.2) | Odisha |
| 114 | Taba | 43284 | 7(70.2) | Arunachal Pradesh | 266 | Salumpicket | Landrace | 7(75.2) | - |
| 115 | Khesra | 43285 | 7(78.2) | Arunachal Pradesh | 267 | IET 21627 | Culture | 7(85.2) | CRRI,Cuttack |
| 116 | Mopu | 43286 | 3(88.2) | Arunachal Pradesh | 268 | IR64 Sub-1 | Culture | 5(81.2) |  |
| 117 | Phondum | 9241 | 5(84.2) | Manipur | 269 | IR20 | Variety | 7(85.2) | IRRI,Philippines |
| 118 | Langme-1 | 43287 | 3(88.2) | Arunachal Pradesh | 270 | IRRI-122 | Culture | 7(87.2) | IRRI,Philippines |
| 119 | Langme-2 | 43288 | 3(86.2) | Arunachal Pradesh | 271 | IR36 | Variety | 7(83.2) | IRRI,Philippines |
| 120 | Sansangba | 9242 | 7(88.2) | Manipur | 272 | Mahulata | 35186 | 7(87.2) | Odisha |
| 121 | Jamek | 43289 | 3(86.2) | Arunachal Pradesh | 273 | Swarna Sub1 | Variety | 7(89.2) | Uttar Pradesh,Odisha |
| 122 | Umbo | 43290 | 3(82.2) | Arunachal Pradesh | 274 | IR64 | Variety | 5(79.2) | Odisha |
| 123 | Shiyas | 9393 | 7(88.2) | Manipur | 275 | IR72 | Variety | 7(87.2) | IRRI,Philippines |
| 124 | Umleng-1 | 43291 | 1(94.2) | Arunachal Pradesh | 276 | Sadabahar | Variety | 9(93.2) | Jharkhand |
| 125 | Charmui | 43292 | 3(86.2) | Arunachal Pradesh | 277 | Naveen | Variety | 7(87.2) | Odisha |
| 126 | Kapongla | 9399 | 7(88.2) | Manipur | 278 | Swarna MAS | Culture | 7(83.2) | CRRI,Cuttack |
| 127 | Taba dugu | 43293 | 3(88.2) | Arunachal Pradesh | 279 | Tapaswini MAS | Variety | 7(79.2) | Odisha |
| 128 | Umleng-2 | 43294 | 3(84.2) | Arunachal Pradesh | 280 | IR64 MAS | Culture | 5(77.2) | CRRI,Cuttack |
| 129 | Angainaora | 9412 | 5(72.2) | Manipur | 281 | Hema | Variety | 9(89.2) | Odisha |
| 130 | Kala Bora | 43295 | 7(76.2) | Arunachal Pradesh | 282 | Parijat | Variety | 7(81.2) | Odisha |
| 131 | Tesang | 43296 | 7(88.2) | Arunachal Pradesh | 283 | Champa | 3399 | 9(87.2) | Odisha |
| 132 | Tsabu | 43297 | 7(70.2) | Arunachal Pradesh | 284 | Pateni | 22134 | 7(89.2) | Odisha |
| 133 | Phouxin | 9424 | 7(88.2) | Manipur | 285 | Patnai | Landrace | 7(75.2) | Odisha |
| 134 | CR3820-4-5-3-4-1 | Culture | 9(88.2) | CRRI,Cuttack | 286 | Khadra | 283026 | 7(85.2) | Odisha |
| 135 | Bamak | 43299 | 3(82.2) | Arunachal Pradesh | 287 | CR1014 | Variety | 7(81.2) | Odisha |
| 136 | Kolong | 43300 | 7(78.2) | Arunachal Pradesh | 288 | Gayatri | Variety | 7(87.2) | Odisha |
| 137 | Jessaria | 9274 | 7(88.2) | Manipur | 289 | Manika | Variety | 7(83.2) | Odisha |
| 138 | Umpa-1 | 43301 | 7(86.2) | Arunachal Pradesh | 290 | Urbasi | Variety | 7(79.2) | Odisha |
| 139 | Kabokphou | 9021 | 7(78.2) | Manipur | 291 | Mrulalini | Variety | 7(65.2) | Odisha |
| 140 | Langmanbi | 9022 | 9(88.2) | Manipur | 292 | Tejaswani | Variety | 7(69.2) | Odisha |
| 141 | Umpa-2 | 43302 | 7(84.2) | Arunachal Pradesh | 293 | Ranidhan | Variety | 7(87.2) | Odisha |
| 142 | Sarpung | 43303 | 9(94.2) | Arunachal Pradesh | 294 | Rambha | Variety | 7(81.2) | Odisha |
| 143 | Moirangphou | 9023 | 7(82.2) | Manipur | 295 | Mandakini | Variety | 7(87.2) | Odisha |
| 144 | Jagam | 43304 | 7(84.2) | Arunachal Pradesh | 296 | Mahanadi | Variety | 7(75.2) | Odisha |
| 145 | Kati khalli | 43305 | 7(78.2) | Arunachal Pradesh | 297 | Surendra | Variety | 7(65.2) | Odisha |
| 146 | Bansmati | 43306 | 7(86.2) | Arunachal Pradesh | 298 | CRDhan 500 | Variety | 7(83.2) | Odisha,UP |
| 147 | Kala joha | 43086 | 9(82.2) | Assam | 299 | Reeta | Variety | 7(81.2) | Odisha |
| 148 | Tenglai | 43087 | 7(88.2) | Assam | 300 | Phalguni | Variety | 7(72.2) | Odisha |
| 149 | Ranjit | 43088 | 7(82.2) | Assam | 301 | Luna Sunkhi | variety | 7(70.2) | Odisha |
| 150 | Mota Dhan | 43089 | 7(88.2) | Assam | 302 | Hazaridhan | variety | 5(76.2) | Jharkhand |
| 151 | Phasi | 43090 | 7(66.2) | Assam | 303 | Gautam | variety | 5(93.2) | Bihar |
| 152 | Ratna | Variety | 7(72.2) | Odisha,Gujarat | 304 | Pyari | Variety | 5(79.2) | Odisha |

**Parentheses contains adjusted mean % of number of plants in a score in response to low temperature stress.**

Critical Differences (Between) SEd CD _5%_

Two Control Treatments 4.4 9.2

Two Test Treatments (Same Block) 10.4 23.1

Two Test Treatments (Different Blocks) 11.2 23.9

A Test Treatment and A Control Treatment 9.1 18.3
