# Supplementary figures and images for: Genome-Wide Association Mapping Reveals Multiple QTLs Governing Tolerance Response for Seedling Stage Chilling Stress in Indica Rice
Source: Front Plant Sci. 2017 Apr 25;8:552. doi: 10.3389/fpls.2017.00552 (PMC5404645; doi:10.3389/fpls.2017.00552)

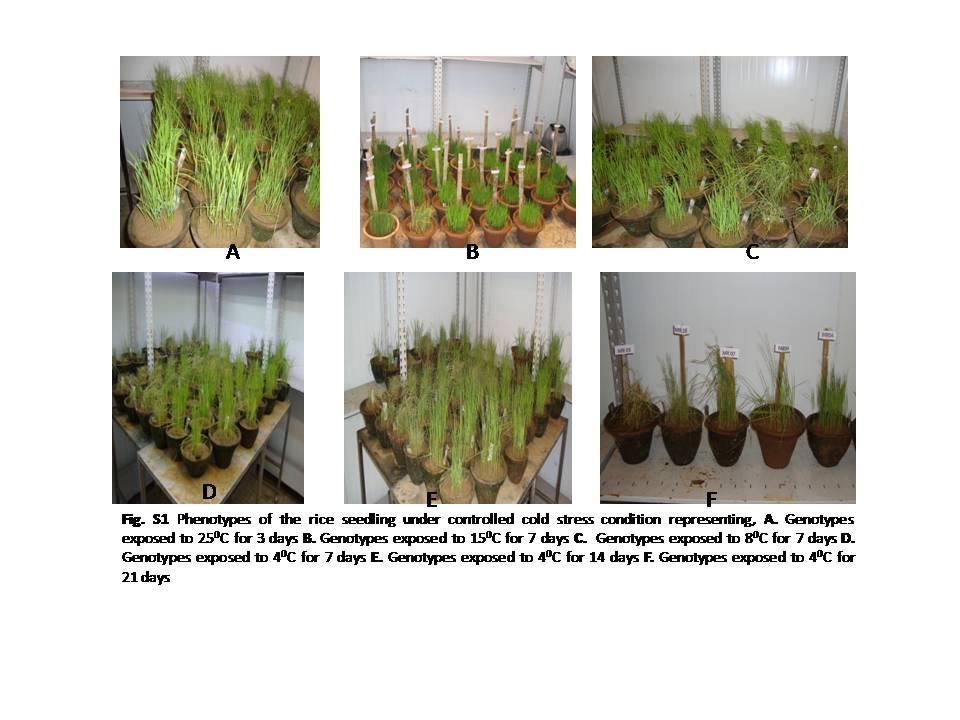

Supplement: Supplementary file 3 [file Image1.JPEG]

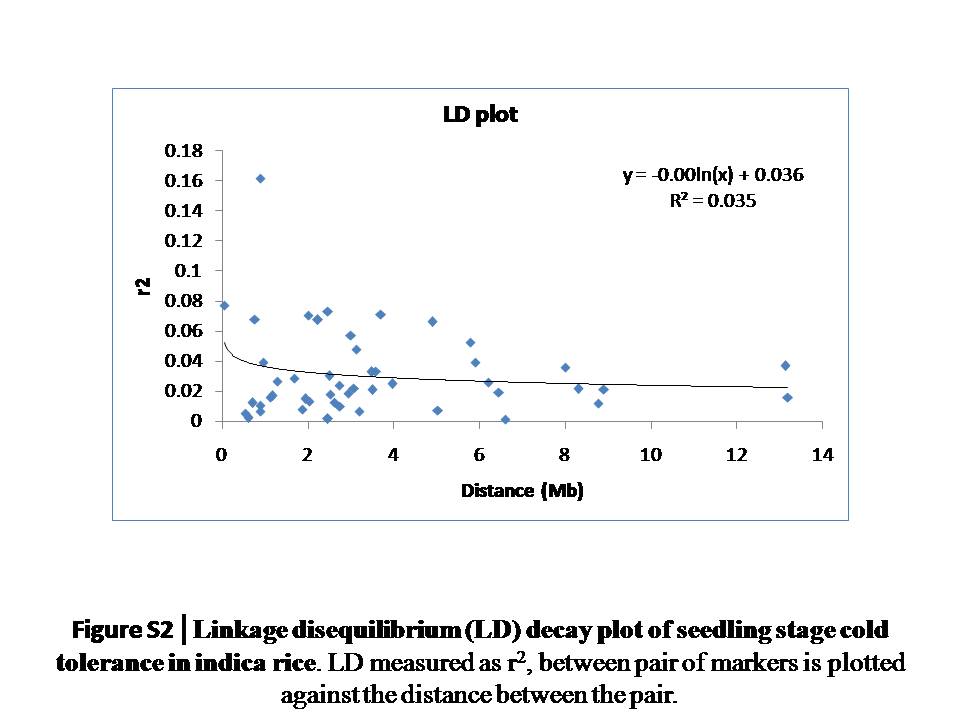

Supplement: Supplementary file 4 [file Image2.JPEG]
